# FR58P1a; a new uncoupler of OXPHOS that inhibits migration in triple-negative breast cancer cells via Sirt1/AMPK/β1-integrin pathway

**DOI:** 10.1038/s41598-018-31367-9

**Published:** 2018-09-04

**Authors:** Félix A. Urra, Felipe Muñoz, Miguel Córdova-Delgado, María Paz Ramírez, Bárbara Peña-Ahumada, Melany Rios, Pablo Cruz, Ulises Ahumada-Castro, Galdo Bustos, Eduardo Silva-Pavez, Rodrigo Pulgar, Danna Morales, Diego Varela, Juan Pablo Millas-Vargas, Evelyn Retamal, Oney Ramírez-Rodríguez, Hernán Pessoa-Mahana, Mario Pavani, Jorge Ferreira, César Cárdenas, Ramiro Araya-Maturana

**Affiliations:** 1 Anatomy and Developmental Biology Program, Institute of Biomedical Sciences, University of Chile, Santiago, Chile; 2 Geroscience Center for Brain Health and Metabolism, Santiago, Chile; 3 Departamento de Química Orgánica y Físico-Química, Facultad de Ciencias Químicas y Farmacéuticas, Universidad de Chile, Casilla 233, Santiago 1, Chile; 4 Laboratorio de Bioinformática y Expresión Génica, INTA-Universidad de Chile, El Líbano, 5524 Santiago, Chile; 5 Programa de Fisiología y Biofísica, Instituto de Ciencias Biomédicas, Facultad de Medicina, Universidad de Chile, Santiago, 8380453 Chile; 6 Millennium Nucleus of Ion Channels-Associated Diseases (MiNICAD), Universidad de Chile, Santiago, Chile; 7 Campus Río Simpson, University of Aysén, Obispo Vielmo 62, Coyhaique, 5952122 Aysén, Chile; 8 Programa de Farmacología Molecular y Clínica, Instituto de Ciencias Biomédicas (ICBM), Facultad de Medicina, Universidad de Chile, Independencia 1027, Casilla 7, Santiago, Chile; 9 Department of Chemistry and Biochemistry, University of California, Santa Barbara, California, 93106 United States; 10 The Buck Institute for Research on Aging, Novato, CA 94945 United States; 11 Instituto de Química de Recursos Naturales and Programa de Investigación Asociativa en Cáncer Gástrico, Universidad de Talca, casilla 747, Talca, Chile

## Abstract

Highly malignant triple-negative breast cancer (TNBC) cells rely mostly on glycolysis to maintain cellular homeostasis; however, mitochondria are still required for migration and metastasis. Taking advantage of the metabolic flexibility of TNBC MDA-MB-231 cells to generate subpopulations with glycolytic or oxidative phenotypes, we screened phenolic compounds containing an *ortho*-carbonyl group with mitochondrial activity and identified a bromoalkyl-ester of hydroquinone named FR58P1a, as a mitochondrial metabolism-affecting compound that uncouples OXPHOS through a protonophoric mechanism. In contrast to well-known protonophore uncoupler FCCP, FR58P1a does not depolarize the plasma membrane and its effect on the mitochondrial membrane potential and bioenergetics is moderate suggesting a mild uncoupling of OXPHOS. FR58P1a activates AMPK in a Sirt1-dependent fashion. Although the activation of Sirt1/AMPK axis by FR58P1a has a cyto-protective role, selectively inhibits fibronectin-dependent adhesion and migration in TNBC cells but not in non-tumoral MCF10A cells by decreasing β1-integrin at the cell surface. Prolonged exposure to FR58P1a triggers a metabolic reprograming in TNBC cells characterized by down-regulation of OXPHOS-related genes that promote cell survival but comprise their ability to migrate. Taken together, our results show that TNBC cell migration is susceptible to mitochondrial alterations induced by small molecules as FR58P1a, which may have therapeutic implications.

## Introduction

Current anticancer therapies target the uncontrolled clonal proliferation of cancer cells, which proves to be a limited strategy for solid tumors in which the proliferation is accompanied by the ability to invade and execute metastasis^1^. Notably, the molecular mechanisms of migration are not inhibited or affected by conventional anti-cancer drugs^2^, converting the search for and design of specific drugs to inhibit migration and invasion of solid cancers into a highly relevant quest^1,3^. Breast cancer is a heterogeneous disease comprised of several biologically distinct sub-types that, despite great progress in the early detection and development of clinical therapy, still is the leading cause of women’s death mainly associated with cancer metastases^4,5^. In particular, triple-negative breast cancer (TNBC) sub-type that lacks the expression of estrogen receptor (ER), progesterone receptor (PR) and human epidermal growth factor receptor (HER2)^6^ is associated with a 4-fold increased risk of distant metastasis and shorter overall survival^7^. Patients with TNBC develop pulmonary, hepatic and cerebral metastases more frequently than other breast cancer sub-types^8,9^ and chemotherapy treatments are limited, highlighting the need to understand the biology of TNBC.

Consistent with the Warburg effect, proliferating, non-metastatic breast cancer cells meet their metabolic demand mainly through glycolysis^10^, although, the contribution of mitochondrial metabolism is still crucial to promote cancer cell survival^11,12^ and tumor adaptation to an unfavorable microenvironments^13^. On the other hand, invasive metastatic breast cancer cells specifically favor mitochondrial respiration, to increase ATP levels, through a mechanism that involves overexpression of PGC-1α and increase mitochondrial biogenesis^14^. In fact, it has been shown that OXPHOS activity increases concomitantly with the metastatic potential in primary breast cancer^15,16^. Therefore, mitochondrial metabolism represents an attractive target for anti-metastatic approaches.

The TNBC cell line MDA-MB-231 is commonly considered a cell line with a high glycolytic rate and a low level of respiration^17,18^. By substituting glucose for galactose, we obtained MDA-MB-231 subpopulations that show a high dependence on respiration and decreased participation of glycolysis to supply energy demand^19^. Under these conditions, we evaluated the effect of phenolic compounds based on hydroquinone, benzofuran, benzophenone and chromone scaffolds containing an *ortho*-carbonyl group on the proliferation of glycolytic and oxidative MDA-MB-231 cell subpopulations and used the results to predict and select compounds with mitochondrial effects for the first time in breast cancer cells. A bromoalkyl ester of a hydroquinone derivative (FR58P1a) was selected through this test and identified as a new protonophoric uncoupler of OXPHOS that induces mitochondrial NADH oxidation, and Sirt1/AMPK-activation that in turn inhibits fibronectin-dependent migration of TNBC cells by decreasing β1-integrin at the cell surface.

## Results

### Bioenergetic profiles of metabolically different subpopulations of MDA-MB-231 breast cancer cells

**Figure 1:**
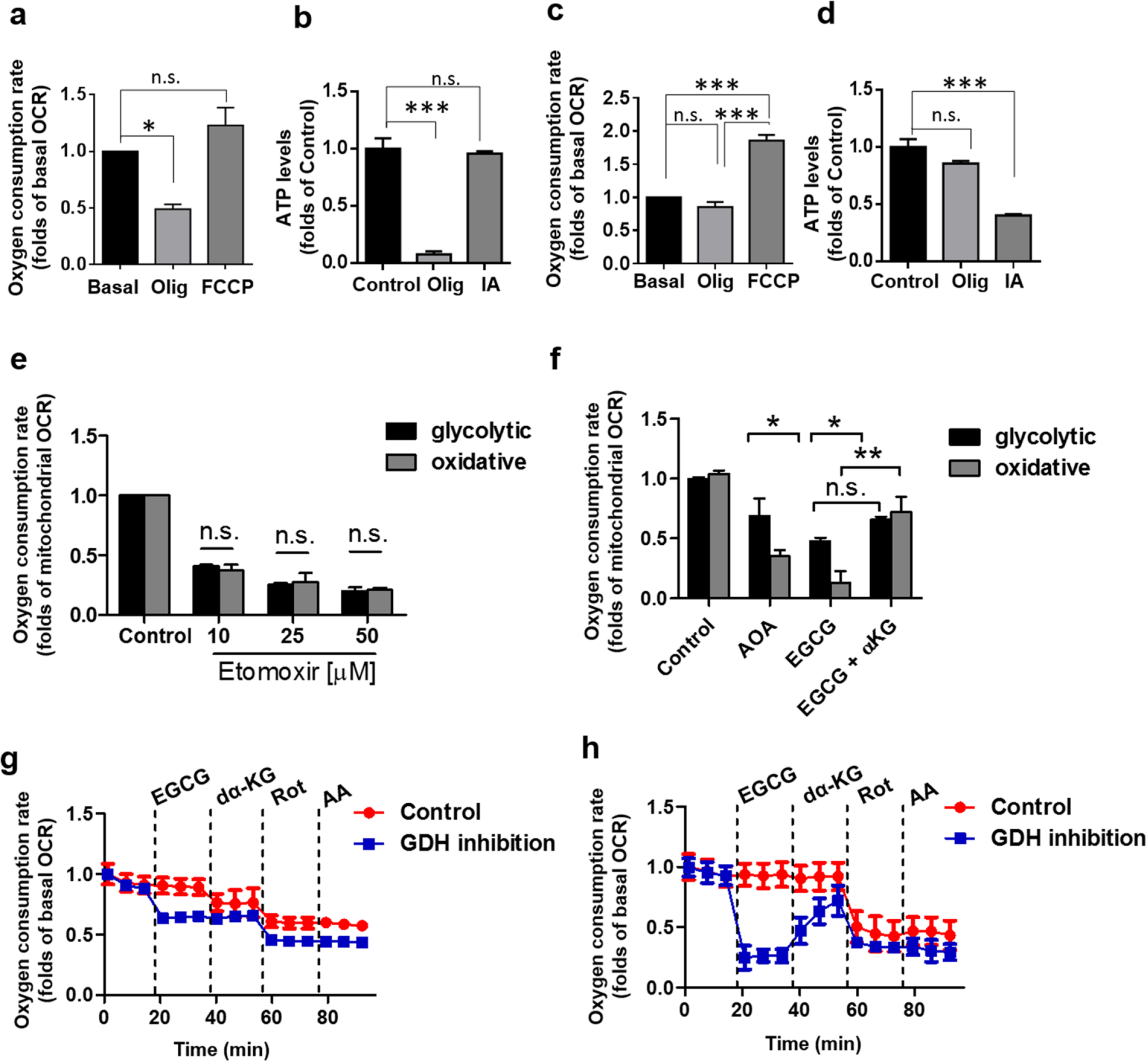
Metabolic differences between oxidative and glycolytic subpopulations of TNBC MDA-MB-231 cells. Energy contribution of mitochondrial respiration and intracellular ATP levels of oxidative (**a**,**b**) and glycolytic (**c**,**d**) subpopulations of TNBC MDA-MB-231 cells. Contribution of fatty acid oxidation (**e**) and glutaminolysis (**f**) to mitochondrial respiration. Inhibition of mitochondrial respiration by GDH inhibitor EGCG (100 µM) and reverse by cell permeable metabolite dimethyl-α-ketoglutarate (1 mM, dα-KG) in glycolytic (**g**) and oxidative (**h**) subpopulations. AOA: aminooxyacetic acid; EGCG: epigallocatechin-monogallate; IA: iodoacetate; Olig.: oligomycin. Data are expressed as means ± SEM of three independent experiments. *p < 0.05, **p < 0.01, ***p < 0,001 vs Control. n.s.: not significant.

Cancer cells exhibit metabolic flexibility that allow them to adapt to metabolic stress^20,21^. By replacing glucose for galactose in the media, leaving glutamine as the main source of carbon by seven days, we generated a population of MDA-MB-231 cells that mainly relies on OXPHOS, as revealed by a high basal mitochondrial respiration and coupling efficiency (Supplementary Fig. S1), that was inhibited by the ATP synthase inhibitor oligomycin (Fig. 1a) causing a drastic drop in the ATP levels (Fig. 1b). On the other hand, the glycolytic subpopulation grown in glucose and glutamine exhibited a reduced basal mitochondrial respiration (Supplementary Fig. S1) and intracellular ATP levels that were unaffected by oligomycin (Fig. 1c,d). The intracellular ATP levels were significantly reduced when the cells were treated with the glycolysis inhibitor iodoacetate (IA) (Fig. 1d). On absence of glucose, mitochondria can use fatty acids and/or amino acids such as glutamine as carbon substrates to maintain OXPHOS^22,23^. Thus, we first determine the role of fatty acids in both subpopulations of MDA-MB-231 by inhibiting fatty acid oxidation with etomoxir (an irreversible inhibitor of carnitine palmitoyltransferase-1) and the effect on basal mitochondrial OCR was evaluated. As shown in Fig. 1e, fatty acid oxidation contributed in a similar extension to mitochondrial respiration in both subpopulations, suggesting that this route is not exclusive for oxidative MDA-MB-231 cells. Then, to determine the role of glutamine, we inhibited both transaminases and glutamate dehydrogenase using aminooxyacetic acid (AOA) and epigallocatechin-monogallate (EGCG), respectively, to prevent the conversion of glutamine-derived glutamate into α-ketoglutarate (αKG)^24^. Both treatments produced a reduction in the mitochondrial OCR in both subpopulations; however, the reduction was stronger in oxidative MDA-MB-231 cells (Fig. 1f). Along these lines, the injection of cell permeable metabolite dimethyl alpha-ketoglutarate only reversed the OCR inhibition produced by EGCG in oxidative MDA-MB-231 cells (Fig. 1g,h). Interestingly, the OCR observed in both cell subpopulations were sensitive to rotenone in a similar extension that antimycin, suggesting that glutaminolysis via GDH/αKG/Complex I in MDA-MB-231 cells in the main route for OXPHOS. No differences in the NAD(P)H, ATP, OGDH and PGC1α levels were detected between both subpopulations (Supplementary Fig. S1e–h).

### Identification of compounds with mitochondrial action using a test based on a shift in energy metabolism

**Figure 2:**
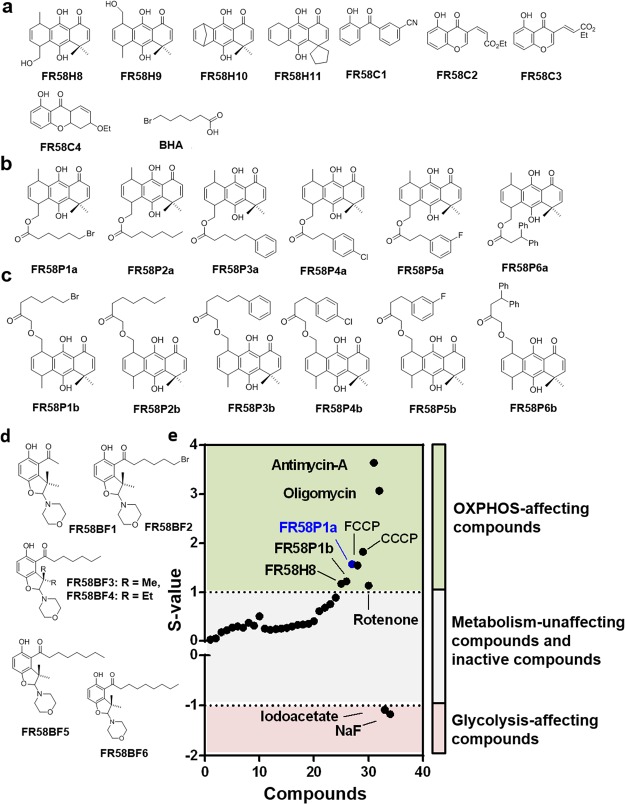
Identification of a new mitochondria-affecting compound. Chemical structures of compounds evaluated (**a**) hydroquinones, benzophenones and chromones (**b**,**c**) alkyl ester of hydroquinone derivatives and (**d**) benzofuranes. (**e**) S-values obtained from metabolic shift screening in subpopulations of MDA-MB-231 cells. BHA: Bromo hexanoic acid, Olig: oligomycin, IA: iodoacetate.

Next, we used the substantial metabolic differences between these two subpopulations of MDA-MB-231 cells to identify from a new series of *ortho*-carbonyl substituted scaffold-containing compounds (Fig. 2a–d; synthesis is showed in Supplementary Scheme 1), which may have mitochondrial action. We evaluated the effect of three groups of phenolic compounds containing an *ortho*-carbonyl group based on hydroquinone (Fig. 2a–c), chromone (Fig. 2a) and benzophenone and benzofuran scaffolds (Fig. 2d) on the proliferation of both oxidative and glycolytic MDA-MB-231 subpopulations at 48 h of exposition. The anti-proliferative effect of these compounds was calculated using the ratio log (IC_50_ in glycolytic cells/IC_50_ in oxidative cells) named as the S-value^25^. This method shows that glycolytic inhibitors (NaF and IA) exhibited strong anti-proliferative effect only in glycolytic cells given S-values <−1 (Fig. 2e and Supplementary Fig. S2a), whereas inhibitors and uncouplers that affect mitochondrial function (antimycin-A, oligomycin, rotenone, FCCP, CCCP) only affected oxidative cells given S-values >1 (Fig. 2e and Supplementary Fig. S2b). Based on this characterization, we found that all benzophenone (Fig. 2a), chromone (Fig. 2a) and benzofuran (Fig. 2d) derivatives were predicted as compounds without mitochondrial action (S-values > 0 and <1) (Fig. 2e and Supplementary Figs S2c,d, S3 and Table S1). Similar results were found in hydroquinone derivative compounds in which the non-phenolic hydroxyl group had been blocked by esterification (Fig. 2b,c, Supplementary S2–S3 and Table S1), with the exception of compound FR58P1a, which had a bromo-substituted alkyl chain in the acyl moiety, that inhibited proliferation only in oxidative cells, exhibiting a S-value of +1.5, similar to the one observed for known mitochondrial uncouplers of OXPHOS such as FCCP and CCCP (Fig. 2e), suggesting that FR58P1a compromises mitochondrial function. The quality of our screening for the identification of OXPHOS-affecting compounds by using the glucose-glutamine/galactose-glutamine method was supported by a Z’ factor = 0.964.

### The bromoalkyl ester of a hydroquinone derivative FR58P1a uncouples OXPHOS and has no effect on complex I

**Figure 3:**
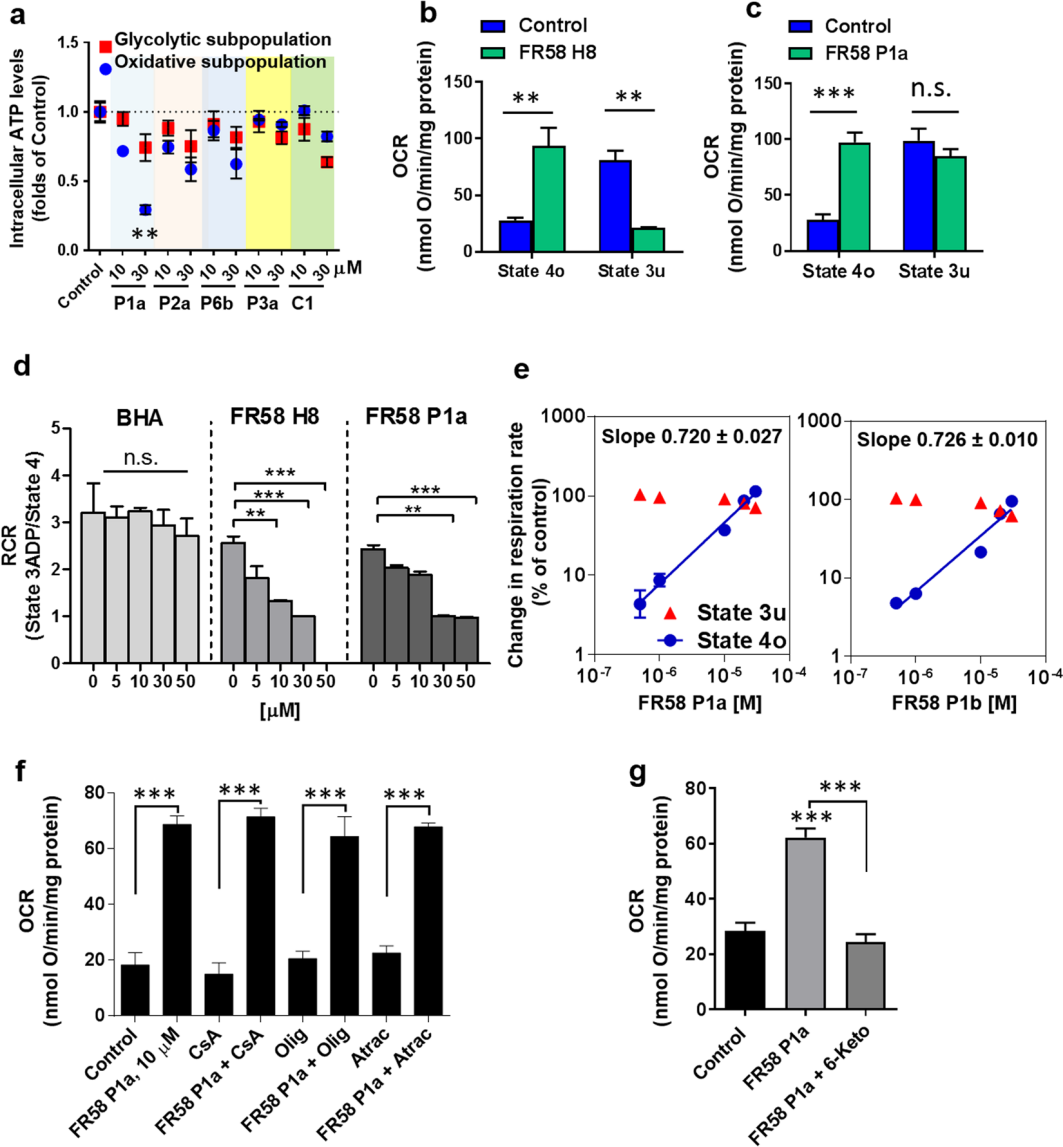
FR58P1a is a protonophore uncoupler of OXPHOS in mitochondria isolated from TA3/Ha cancer cells. (**a**) Effect of predicted inactive compounds and FR58P1a on ATP levels in glycolytic (red points) and oxidative (blue points) subpopulations of MDA-MB-231 breast cancer cells at 4 h of exposure. (**b**,**c**) Effect of FR58H8 and FR58P1a on complex I-dependent respiration (stimulated with glutamate plus malate) in state 4o and 3 u, in presence of 1 µM oligomycin and 0.2 µM CCCP, respectively. (**d**) Effect of bromohexanoic acid (BHA), FR58H8 and FR58P1a on Respiratory Control Ratio (RCR) in presence of glutamate plus malate (G + M), which was calculated as the respiration in state 3ADP divided by that in state 4. (**e**) Double logarithmic plots of uncoupling effect of both regioisomers FR58P1a and P1b in mitochondria isolated from TA3/Ha cells. In blue circles, the effect of FR58P1a and FR58P1b on mitochondrial respiration in state 4o (1 µM oligomycin addition); in red circles, effect of compounds on maximum electron flux (state 3 u by 0.2 µM CCCP addition). Lines were fitted by regression of log-log plot, obtaining the slope log-log plot and K_0.5_ values. (**f**) Dependence of electron transport chain, mPTP, ANT, FoF1-ATP synthase and (**g**) membrane fluidity in the uncoupler effect of FR58P1a. Data shown are the mean ± SEM of three independent experiments. *p < 0.05, **p < 0.01, ***p < 0.001, vs. Control (DMSO). n.s. not significant.

To determine whether FR58P1a has an effect on mitochondrial function, we treated both MDA-MB-231 subpopulations, with 30 µM FR58P1a, a concentration at which an evident effect on proliferation is observed (Supplementary Fig. S2), and the levels of ATP were determined. As predicted, FR58P1a produced a significant decrease of ATP levels in the oxidative cellular subpopulation while the glycolytic subpopulation was unaffected (Fig. 3a). Predicted inactive compounds included in this assay show no effects on ATP levels, confirming the specificity of FR58P1a on mitochondrial metabolism (Fig. 3a). FR58P1a contains the hydroquinone scaffold FR58H8, which has a known effect on complex I-dependent respiration^26^, and a bromo hexanoyl (BHA) tail. To determine whether the effect of FR58P1a is mediated by these compounds after a potential intracellular hydrolysis, we isolated mitochondria from the highly oxidative TA3/Ha cells^26^ and the effects of FR58H8, FR58P1a and BHA on respiration were determined. When we compared the effect of FR58H8 and FR58P1a on respiration in state 3 u (in presence of FCCP) and state 4o (in presence of oligomycin), we found that both compounds increase mitochondrial respiration stimulated with glutamate/malate in state 4o, but only FR58H8 completely inhibited respiration in state 3 u at 50 µM (Fig. 3b,c). Consistently, the mitochondrial respiratory control ratio (RCR) in presence of ADP was decreased close to 1 at 30 µM by both compounds, suggesting an uncoupling of OXPHOS and at 50 µM, FR58H8 produced a RCR value close to 0 (Fig. 3d), which corresponds to the expected inhibition of complex I-dependent respiration (Supplementary Fig. 4a). FR58P1a did not affect the mitochondrial respiration in state 3_ADP_ in a wide range of concentrations (10–100 µM) but the hydroquinone FR58H8 exhibited an inhibitory effect between 50–100 µM. FR58P1a demonstrated a sustained effect stimulating the mitochondrial respiration in State 4o. In this condition, FR58H8 exhibited a bell-shaped curve, inhibiting the OCR at 100 µM (Supplementary Fig. 4b). On the other hand, BHA did not exhibit any effects on RCR (Fig. 3d). In summary, these results show that FR58H8 is a compound with dual activity, producing uncoupling of OXPHOS and inhibiting complex I-dependent respiration while FR58P1a only uncouples OXPHOS, suggesting that the esterification of the hydroxymethyl group at carbon-5 in FR58P1 with BHA blocks the inhibitory action of the parental hydroquinone FR58H8 on complex I, making the effect of FR58P1a on OXPHOS different to other structurally-related hydroquinones. Finally, we determined that FR58P1b, a regioisomer of FR58P1a, also increases the proton leak without affecting the mitochondrial maximum electron flux and both compounds exhibited similar uncoupling activity with K_0.5_ values of 11.10 ± 1.34 µM and 15.91 ± 0.32 µM, respectively (Fig. 3e). We conclude that esters of FR58H8 lose their effect on mitochondrial function, but the bromo alkyl ester substitutions (e.g. FR58P1a and FR58P1b) produce uncouplers of OXPHOS without a direct interaction with ETC, which is a stereochemical-independent effect.

### FR58P1a is a protonophore uncoupler of OXPHOS

Our previous results suggest that FR58P1a has an uncoupling effect on mitochondria. In order to determine the mechanism of uncoupling of OXPHOS produced by FR58P1a, we inhibited known proteins involved in the uncoupling of mitochondria such as the permeability transition pore (PTP) with cyclosporine A, the F_0_F_1_-ATP synthase with oligomycin and the adenine nucleotide translocator (ANT) with atractyloside and state 2 OCR was determined. As shown in Fig. 3f, no effect on the OCR induced by FR58P1a was observed. Then, to determine whether the uncoupling of mitochondria was mediated by protonophoric action of FR58P1a, we treated the isolated mitochondria with 6-ketocholestanol (6-Keto), a keto-derivative of cholesterol that decreases the membrane fluidity preventing uncoupling mediated by protonophores^27,28^. As shown in Fig. 3g, the presence of 6-Keto totally prevents the increase of OCR induced by FR58P1a supporting the notion that FR58P1a uncouples OXPHOS by a protonophoric mechanism.

### FR58P1a induces a mild uncoupling of OXPHOS, affecting bioenergetics and mitochondrial network organization in intact breast cancer and normal cells

**Figure 4:**
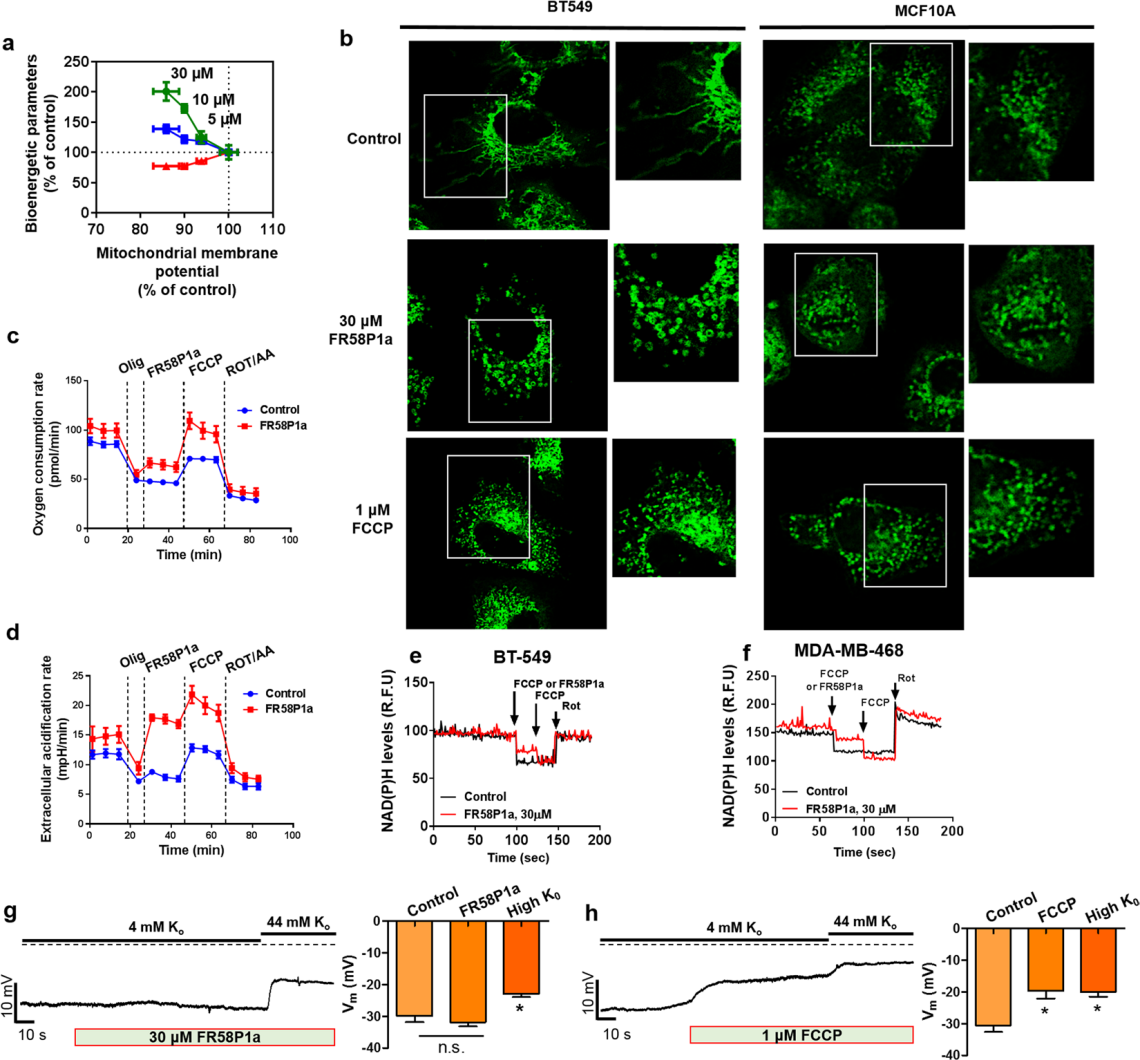
FR58P1a induces mild uncoupling of OXPHOS without effect on plasma membrane potential. (**a**) Effect of FR58P1a on flux-force relationship of bioenergetics parameters in MDA-MB-231 cells, cellular respiration (green square), mitosox fluorescence (blue square), intracellular ATP levels (red square), (**b**) FR58P1a and FCCP produce fragmentation of mitochondrial network of TNBC BT549 and non-tumoral MCF10A cells at 4 h of exposition. (**c**,**d**) FR58P1a produces mild uncoupling of OXPHOS, increasing the TCA activity-dependent ECAR value in MDA-MB-231 cells. (**e**,**f**) FR58P1a induces a mild mitochondrial NADH oxidation in TNBC cells. (**g**) Whole cell voltage clamp recording from MDA-MB-231 cells exposure to 30 µM FR58P1a and (**h**) 1 µM FCCP. Data shown are the mean ± SEM of three independent experiments. *p < 0.05, **p < 0.01, ***p < 0.001, vs. Control (DMSO).

Using intact MDA-MB-231 cells, we evaluate the effect of FR58P1a injection on mitochondrial respiration and Δψ_m_. As shown in the mitochondrial flux-force diagram in Fig. 4a, an increase in the OCR was accompanied by a reduction in the Δψ_m_. Increased superoxide production and NADH oxidation also correlate with the mitochondrial depolarization (Fig. 4a). The loss of Δψ_m_ and the induction of non-selective metabolic stress had been shown to induce mitochondrial fragmentation^29^. Thus, we determine whether FR58P1a has any effect on mitochondrial morphology in BT549 breast cancer cells and in no-tumorigenic MCF10A epithelial breast cells after 4 h of exposure. As shown in Fig. 4b, FR58P1a induces a progressive budding of the mitochondrial network and apparition of ring-like structures in both cell lines (Fig. 4b). Similar structural changes in the mitochondrial network were induced by 1 µM FCCP.

To compare the ability of FR58P1a to stimulate mitochondrial respiration with the well-known protonophore FCCP, we determine OCR and the extracellular acidification rate (ECAR) in MDA-MB-231 cells using a media without glucose, but containing galactose and glutamine. In this condition, the ECAR does not represent the glycolytic activity and can be an indirect determination of TCA cycle activity^30^. This occurs by CO_2_ production in the mitochondrial matrix and consequent, dissociation in HCO^3−^ + H^+^ in the medium, producing acidification of assay medium which is sensitive to electron transport chain inhibitors (rotenone and antimycin A). As expected, after inhibition of ATP-linked respiration by oligomycin, injection of 30 µM FR58P1a induces a discreet but significant rise in both OCR and ECAR that was further increased by the injection of FCCP, reaching a new maximal OCR and ECAR. This new maximum in OCR and ECAR was decreased after the addition of rotenone/antimycin-A (Fig. 4c,d), suggesting that the increase in both OCR and ECAR is caused by stimulation of the TCA cycle activity. Along these lines, FR58P1a shows a mild effect on NADH oxidation in TNBC BT549 and MDA-MB-468 cells compared with the maximal mitochondrial NADH oxidation induced by FCCP (Fig. 4e,f). These data demonstrate that FR58P1a is a protonophore that induces a mild OXPHOS uncoupling in intact cells.

### FR58P1A is a mitochondrial uncoupler that does not depolarize the plasma membrane

Most protonophore uncouplers have off-target activity at other membranes, leading to undesired effects such as plasma membrane depolarization^31–33^. Thus we evaluated whether FR58P1a depolarized the plasma membrane of MDA-MB-231 cells. Surprisingly, in contrast to FCCP, FR58P1a does not affect the plasma membrane (Fig. 4g,h). These results suggest that FR58P1a does not exhibit the adverse plasma membrane effects that may contribute to non-selective cellular effects.

### FR58P1a-induced OXPHOS uncoupling activates cyto-protective Sirt1-AMPK signaling in TNBC cells

**Figure 5:**
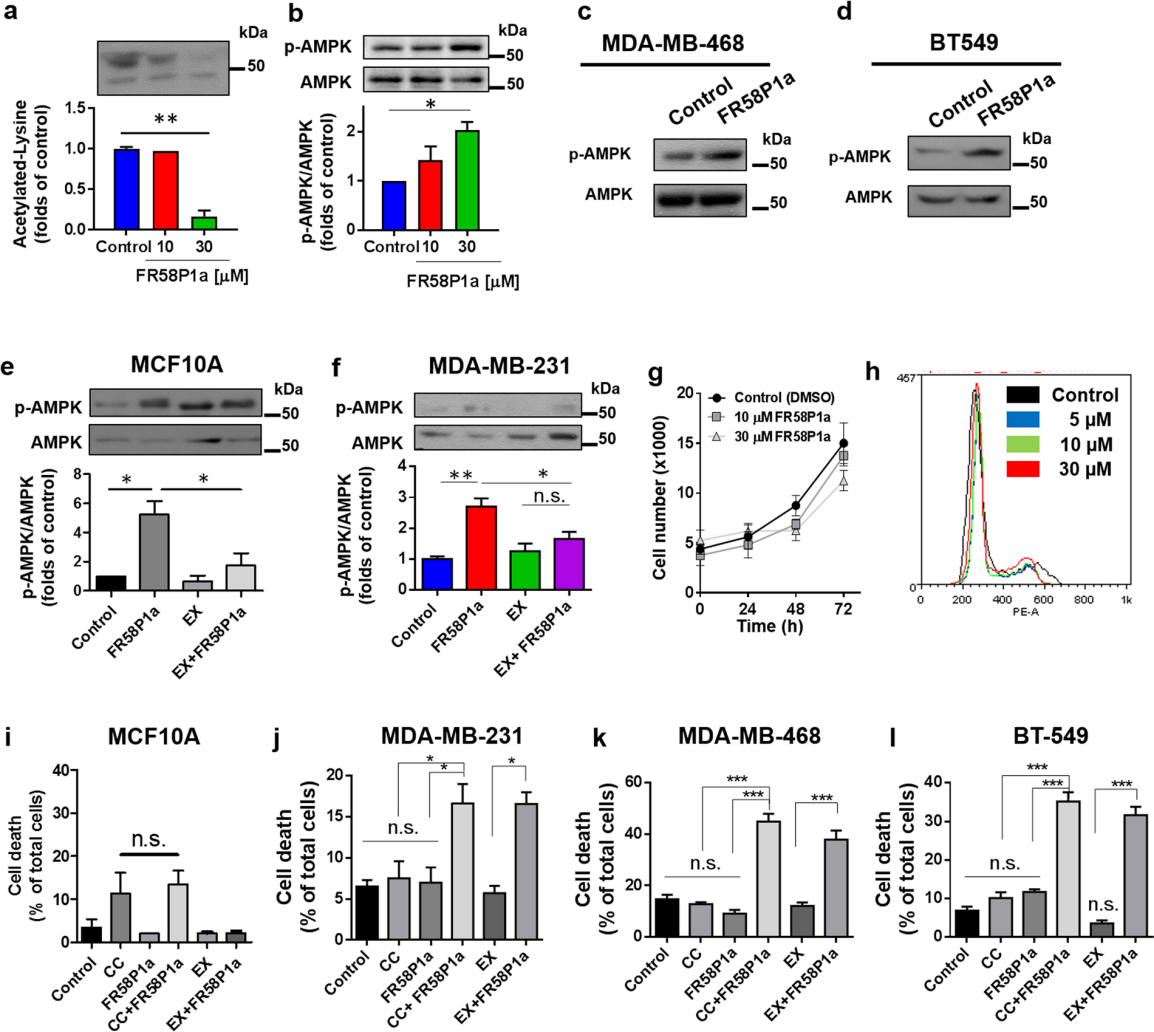
FR58P1a-induced mitochondrial dysfunction activates a cytoprotective Sirt1/AMPK signaling in TNBC cells. (**a**) Levels of intracellular acetylated-lysine and (**b**–**d**) phospho-AMPK levels induced by FR58P1a at 4 h of exposure in TNBC MDA-MB-231 cells, MDA-MB-468 and BT549. (**e**,**f**) Effect of Sirt1 inhibition with 10 µM EX-527 (EX) on phospho-AMPK levels induced by FR58P1a in MCF10A and MDA-MB-231 cells, (**g**,**h**) FR58P1a does not affect the proliferation and cell cycle progression in TNBC MDA-MB-231 and BT-549 cells, respectively, (**i**–**l**) Effect of FR58P1a (30 µM) on MCF10A and TNBC cell death under Sirt1 and AMPK inhibition, with EX-527 and Compound C (CC), respectively at 48 h of exposition. Data shown are the mean ± SEM of three independent experiments. *p < 0.05, **p < 0.01, ***p < 0.001, vs. Control (DMSO). n.s. not significant.

Although most cancer cells *in vivo* greedily uptake glucose driving glycolysis as the main bioenergetic pathway^34^, we and others have demonstrated that mitochondria are still essential for cancer development and progression^11,35^. Thus, we determined the effect of FR58P1a in MDA-MB-231 cells grown in normal culture conditions, which rely heavily on glycolysis^36^. At the three concentrations used (5, 10, 30 µM) FR58P1a induces, as expected, loss of mitochondrial membrane potential after 1 h of treatment (Supplementary Fig. 5a); however, only 30 µM FR58P1a generates a significant decrease of ATP levels as early as 30 min after the treatment, which tends to recover without reaching control levels at 4 h (Supplementary Fig. 5b). Concomitantly, a sustained depletion of intracellular NAD(P)H levels was observed (Supplementary Fig. 5c). In non-tumoral MCF10A cells, FR58P1a increased the intracellular ATP levels and 2NBDG uptake, a fluorescent glucose analogue, at 4 h of treatment, suggesting a remodeling towards glycolysis (Supplementary Fig. S5d,e). As the mitochondrial NADH oxidation is a primary event in the bioenergetic alterations induced by FR58P1a in TNBC cells, we speculate that the decrease in NAD(P)H levels may increase the NAD^+^/NADH ratio, activating deacetylases such as Sirtuin 1 (Sirt1). Accordingly, we evaluate the protein acetylation status of MDA-MB-231 cells treated with FR58P1a during 4 h, finding a significant reduction in the acetylation levels (Fig. 5a). Moreover, the bioenergetic alterations induced by FR58P1a activate AMPK, the main metabolic sensor of the cell^37^, as determined by an increase in its phosphorylation state after 4 h of treatment in TNBC MDA-MB-231 (Fig. 5b), MDA-MB-468 (Fig. 5c) and BT549 (Fig. 5d) cells. Sirt1, which can modulate AMPK activation^37,38^, has been shown to suppresses breast cancer cell grown^39^ and epithelial-to-mesenchymal transition in cancer metastasis^40^. Thus, we determined the levels of AMPK phosphorylation after FR58P1a incubation in MCF10A and MDA-MB-231 cells pre-treated with the Sirt1 inhibitor EX-527. Surprisingly, as shown in Fig. 5e,f, the presence of the Sirt1 inhibitor completely abolishes the activation of AMPK, suggesting a tandem activation of Sirt1 and AMPK under uncoupling of OXPHOS by FR58P1a. At 4 h of treatment with FR58P1a, Sirt1 inhibition produced a greater decrease in the ATP levels, it decreased the 2NBDG uptake and the Δψ_m_ was sensitive to oligomycin, suggesting these data a compensatory role of glycolysis mediated by Sirt1 to maintain the mitochondrial membrane potential by a possible ATPase action (Supplementary Fig. S5f–h). AMPK activation can promote cyto-protection which could explain the lack effect of FR58P1a on viability (Supplementary Fig. S6), proliferation (Fig. 5g) and cell cycle progression (Fig. 5h and Supplementary Fig. S6) in TNBC cells. To prove this hypothesis, we treated TNBC cells with FR58P1a in presence of the AMPK inhibitor compound C (CC) which significantly increases cell death (Fig. 5j–l). Similar results were observed in the presence of Sirt1 inhibitor EX-527. Interestingly, FR58P1a induces Sirt1-dependent AMPK activation in non-tumoral MCF10A breast cells; however, no cell death was observed after the inhibition of either protein with EX-527 or CC at 48 h (Fig. 5i). All together, these results suggest that OXPHOS uncoupling induced by FR58P1a activates the cyto-protective Sirt1-AMPK signaling node that maintains proliferation of TNBC cells, an event no observed in non-tumoral MCF10A cells.

### Sirt1-AMPK activation by FR58P1a inhibits fibronectin-dependent migration in TNBC cells

**Figure 6:**
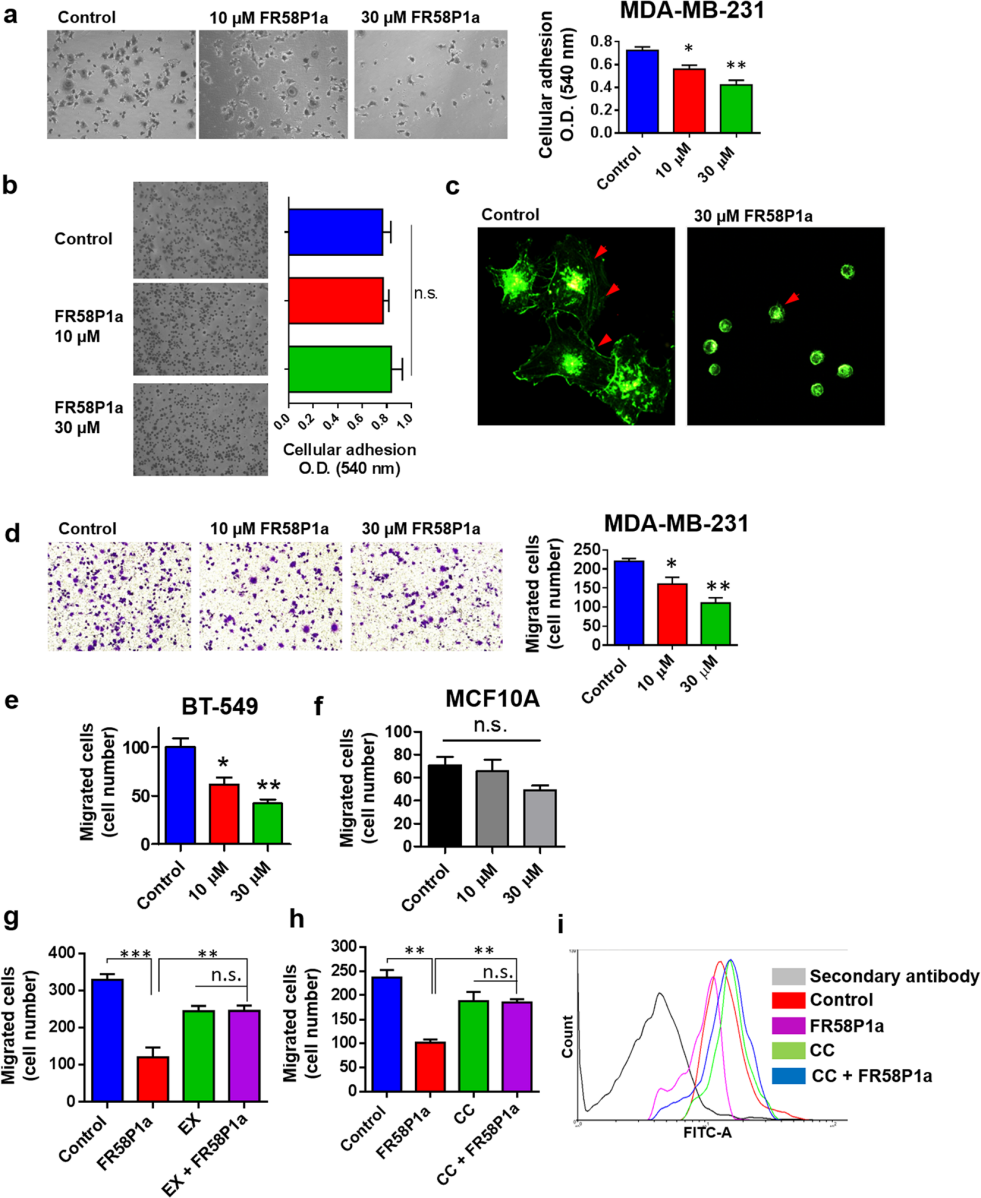
R58P1a inhibits the fibronectin-dependent adhesion and migration by Sirt1/AMPK/β1-integrin axis in TNBC MDA-MB-231 cells. (**a**) Effect of FR58P1a on fibronectin-stimulated adhesion, (**b**) polylysine-stimulated adhesion and (**c**) fibronectin-stimulated actin network of MDA-MB-231 cells. (**d**) Effect of FR58P1a on fibronectin-stimulated migration of MDA-MB-231, (**e**) BT549 and (**f**) MCF10A. (**g**,**h**) Effect of AMPK and Sirt1 inhibition on anti-migratory effect of FR58P1a. (**i**) β1-integrin levels in surface of MDA-MB-231 exposed to FR58P1a and AMPK inhibition with Compound C. Data shown are the mean ± SEM of three independent experiments. *p < 0.05, **p < 0.01, ***p < 0.001, vs. Control (DMSO). n.s. not significant.

In addition to serving as a source of energy and metabolites for proliferating cancer cells^11,36,41,42^, mitochondria have been described as essential during migration and metastasis in breast cancer cells^15,16^. During migration and metastasis, cancer cells must adhere to the extracellular matrix (ECM)^43^. Therefore we evaluated the effect of FR58P1a on adhesion to fibronectin, a non-collagenous ECM glycoprotein essential during invasion and metastasis^44^. At 4 h of exposure to FR58P1a, MDA-MB-231 cancer cells showed a significant reduction of adhesion to fibronectin compared with control (Fig. 6a). Notably, no effects on adhesion to poly-lysine (mediated by an electrostatic charge) were observed (Fig. 6b), suggesting that FR58P1a specifically modifies fibronectin signaling-dependent migration. Along these lines, FR58P1a-treated MDA-MB-231 cells show alterations in the actin network structure (red arrows in Fig. 6c). Then, we evaluated whether OXPHOS uncoupling produced by FR58P1a affects migration in a transwell assay. As shown in Fig. 6d–f, 4 h of exposure to FR58P1a significantly reduces the number of TNBC MDA-MB-231 and BT549 migrating cells without significantly affecting the migration of MCF10A cells (Fig. 6f).

In TNBC cells, the anti-migratory effect of FR58P1a was a ROS-independent event (Supplementary Fig. S7a–d). In order to evaluate if the OXPHOS uncoupling-dependent activation of Sirt1/AMPK axis is involved in the reduction of migration, MDA-MB-231 were incubated with EX-527 and CC for 1 h and FR58P1a for 4 h and the effect on migration was evaluated. CC did not affect basal levels of AMPK phosphorylation, but reduced FR58P1a-induced AMPK activation (Supplementary Fig. S7e). The reduction in migration induced by FR58P1a was reverted by both EX-527 and CC (Fig. 6g,h), indicating that the anti-migratory effect of this compound is Sirt1/AMPK dependent. Similar effects on migration were observed in BT-549 and MDA-MB-468 cells (Supplementary Fig. S7f,g), showing that the mechanism of action of FR58P1a is a general phenomenon, applicable to other TNBC cells. Finally, it is recognized that AMPK activation alters the endomembrane traffic of cell surface proteins, one of them being β1-integrin, a key protein involved in fibronectin-stimulated cell migration^45^. Consequently, we evaluated the effect of FR58P1a on the surface abundance of β1-integrin in MDA-MB-231 cells in presence of fibronectin by flow cytometry. As shown in Fig. 6i, FR58P1a reduced the abundance of β1-integrin compared to control and this was prevented when MDA-MB-231 cells were pre-incubated with CC. Altogether, our data show that the activation of AMPK and Sirt1, induced by mitochondrial dysfunction, impairs cell migration by reducing β1-integrin on the cell surface and in turn reduces cellular adhesion to the ECM.

### FCCP inhibition of fibronectin-dependent migration is not cancer-selective

Using TNBC MDA-MB-231 and BT549 cells and non-tumoral MCF-10A cells, we evaluate the effect of FCCP on the fibronectin-dependent migration during 4 h of treatment. All cell lines treated with 0.5 and 1 μM FCCP exhibited decreased migratory potential (Supplementary Fig. S8a). Then, we determined the levels of AMPK phosphorylation after FCCP incubation in MDA-MB-231 cells pre-treated with the Sirt1 inhibitor EX-527. The presence of the Sirt1 inhibitor completely abolishes the FCCP-induced AMPK activation, suggesting a tandem activation of Sirt1 and AMPK. Furthermore, the reduction in migration induced by FCCP was reverted by the presence of Sirt1 inhibitor in MDA-MB-231 cells (Supplementary Fig. S8b,c). Altogether, our data show that FCCP inhibits non-selectively the fibronectin-dependent migration in TNBC and normal cells, suggesting some secondary mechanism that could be affecting migratory potential in non-tumoral cells.

### Prolonged FR58P1a treatment triggers metabolic adaptation but maintained the inhibitory effect of fibronectin-dependent migration in TNBC cells

**Figure 7:**
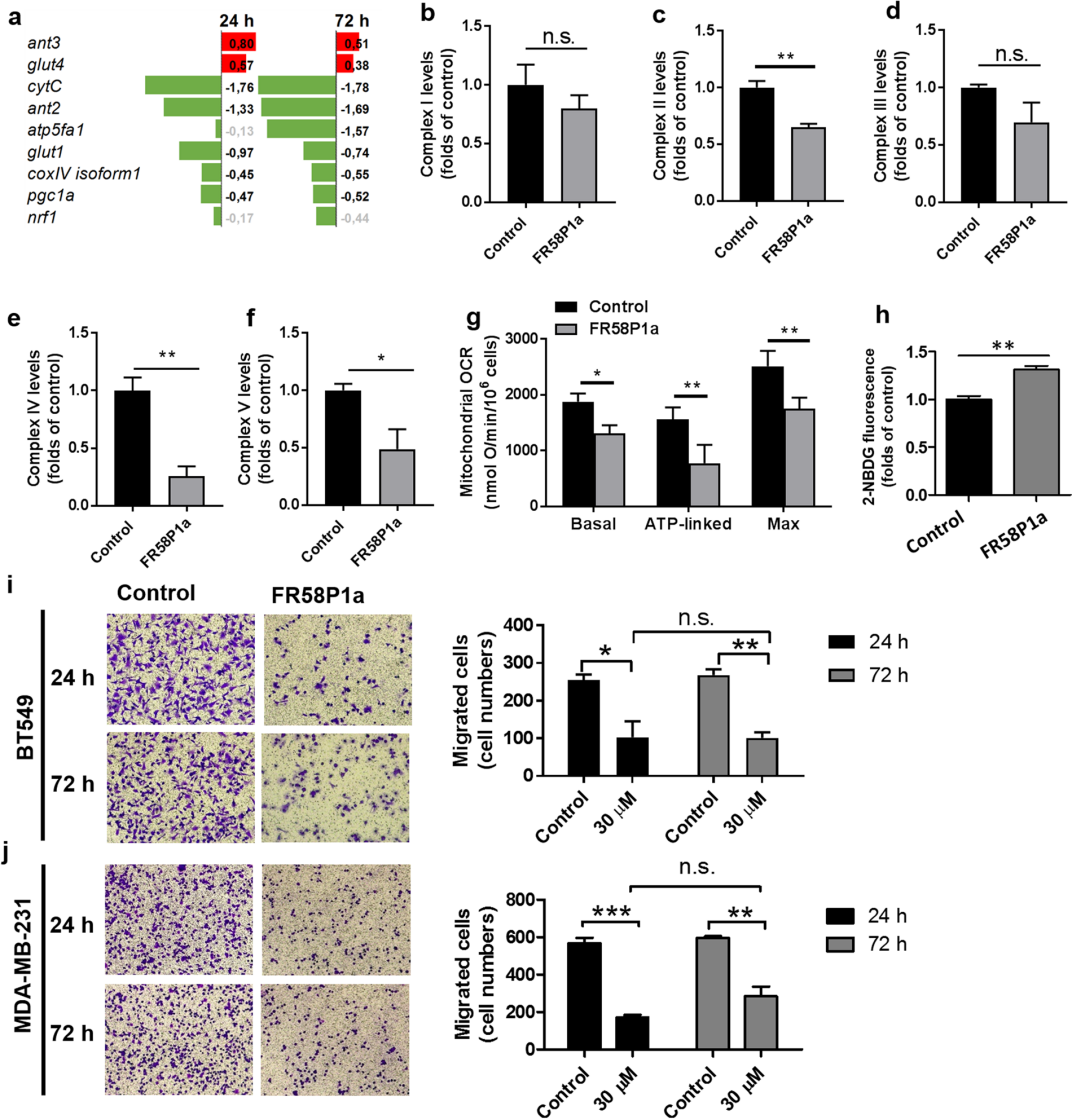
Prolonged FR58P1a treatment triggers metabolic adaptation mediated by mitochondrial clearance, maintaining the inhibitory effect of TNBC cell migration. (**a**) Changes in the expression of genes involved in the OXPHOS, ADP/ATP transport, mitochondrial biogenesis and glycolysis. The bar chart shows the mean log2(fold change) with p-value ≤ 0.05. In green, genes down-regulated and in red genes that are upregulated. Genes without changes are shown in gray. (**b**–**f**) Levels of respiratory complexes. (**g**,**h**) Effect of FR58P1a on mitochondrial oxygen consumption rate and 2-NBDG uptake in MDA-MB-231 cells treated by 24 h. (**i**,**j**) Effect of FR58P1a on fibronectin-dependent migration in TNBC cells treated by 24 and 72 h. Data shown are the mean ± SEM of three independent experiments. *p < 0.05, **p < 0.01, ***p < 0.001, vs. Control (DMSO). n.s. not significant.

It has been recognized that OXPHOS uncoupling, induced by protonophores or ectopic expression of uncoupling protein 1 (UCP1), produces morphological changes in the mitochondria associated with mitochondrial biogenesis and metabolic adaptation toward oxidative metabolism *in vivo* and *in vitro*^46–48^. Therefore, we evaluate whether a prolonged FR58P1a-induced OXPHOS uncoupling in TNBC cells triggers adaptation toward oxidative metabolism promoting enhancing instead of inhibiting fibronectin-dependent migration. The MDA-MB-231 cells were treated with 30 µM FR58P1a for 24 and 72 h and the expression levels of nine genes involved in OXPHOS (*cox-iv isoform 1*, *cyt c*, *atp5fa1*), mitochondrial ADP/ATP transport (*ant2*, *ant3*), mitochondrial biogenesis (*pgc1a*, *nfr-1*) and glucose transport (*glut1*, *glut4*) was evaluated. At 24 and 72 h, *ant3* and *glut4* genes were up-regulated and the OXPHOS-related genes were down-regulated (Fig. 7a). Consistent with this, MDA-MB-231 cells treated with FR58P1a for 24 h exhibited reduced levels of respiratory complexes II, IV and V compared with control condition (Fig. 7b–f), reduced content of cardiolipin, the main phospholipid of mitochondrial membranes^49^ (Supplementary Fig. S9a), and reduced levels of mitochondrial outer membrane proteins VDAC and TOM20 (Supplementary Fig. S9b). These results suggest a decrease in the number of mitochondria. To evaluate if a process of mitochondrial autophagy is involved in the metabolic adaptation of MDA-MB-231 cells to mild uncoupling of OXPHOS by FR58P1a, we evaluate the levels of PINK, a mitophagy marker^50,51^ at 24 of treatment. As Supplementary Fig. 9b shows, FR58P1a produces an increase in PINK1 levels. Consistent with mitophagy^52,53^, these TNBC cells also shown reduced mitochondrial OCR (Fig. 7g) with increased 2NBDG uptake (Fig. 7h), indicating that FR58P1a-treated MDA-MB-231 cells exhibit a metabolic switch towards glycolysis. Remarkably, the inhibitory effect on fibronectin-dependent migration in TNBC MDA-MB-231 and BT549 cells remains unchanged after 24 and 72 h of treatment (Fig. 7i,j). Taken together, these results indicate that mild OXPHOS uncoupling induced by FR58P1a triggers a metabolic adaptation toward glycolysis, involving a mitochondrial clearance, that promotes cancer cell survival but decreases the migratory capability of TNBC cells.

## Discussion

Triple-negative breast cancer (TNBC) cells exhibit a high metastatic potential and lack of standard pharmacologic therapy^5,7^, affecting the overall survival of women with this breast cancer sub-type^54^. These considerations highlight the need to understand the biology of TNBC cells and to search for novel targets for anti-cancer therapies.

Here, based on the high metabolic plasticity of MDA-MB-231 cell line, we generated two subpopulations (glycolytic and oxidative) that we used to screen anti-cancer compounds with mitochondrial action for the first time in breast cancer cells. Previously, we described an anti-cancer *ortho*-carbonyl substituted hydroquinone scaffold^55^ that through small structural changes determines three types of compounds with mitochondrial action^26^. One of these types corresponds to compounds with dual action (inhibitors of complex I-dependent respiration and uncouplers of OXPHOS) whose ester derivatives and benzophenone, chromone and benzofuran scaffolds were used to screen the effect on these cells. To obtain fast data interpretation, we used a nutrient-sensitized index, S-value, described by Gohil *et al*.^25^. This S-value allows to determine whether a compound affects either glycolysis or OXPHOS, by comparing their effects with known metabolic inhibitors. Using this approach we identified a bromoalkyl ester of hydroquinone derivative (FR58P1a) with high positive S-value (+1.5), indicative of specific action on mitochondrial metabolism, similar to what was observed for known OXPHOS uncouplers (Supplementary Table 1). No effects were observed with the other compounds containing *ortho*-carbonyl substitutions (Supplementary Table 1 and Supplementary Fig. S2).

Although the induction of OXPHOS uncoupling has been recognized as a possible therapeutic strategy for obesity^46,56^, neurodegeneration^57,58^, aging^59^, renal ischemia/reperfusion injury^60^, heart injury^61^ and cancer^62,63^, well-known protonophore OXPHOS uncouplers exhibit a narrow therapeutic window^64^ and off-target effects at other membranes, producing toxicity^31–33^ and limiting their potential clinical uses. In contrast to FCCP, FR58P1a does not depolarize the plasma membrane and induces a mild OXPHOS uncoupling, inducing mitochondrial NADH oxidation with sustained mitochondrial depolarization and a transient ATP decrease that activates AMPK in a Sirt1-dependent fashion. Interestingly, the activation of Sirt1/AMPK axis by FR58P1a has a cyto-protective effect in TNBC cells, promoting cell survival and proliferation but inhibiting their ability to migrate. In non-tumoral MCF10A cells, the activation of Sirt1/AMPK axis by FR58P1a only induces an early metabolic remodeling toward glycolysis to maintain ATP levels as previously reported^65,66^, lacking pro-survival and anti-migratory effects. These results suggest that possible aberrant metabolic signaling accompanies Sirt1/AMPK activation in TNBC cells.

The role of AMPK activation during migration in cancer cells is controversial. AMPK sustains migration by promoting the trafficking of mitochondria to the leading edge^67^ and on the other hand, can suppress migration by attenuating lamellipodia formation through the suppression of Rac1-Arp2/3 signaling pathway^68^, and by reduction of β1-integrin membrane traffic^45^. Regarding breast cancer, samples from patients exhibit reduced AMPK signaling, which correlates with high axillary node metastasis^69^, suggesting that AMPK re-activation may have therapeutic implications in breast cancer. Consistent with this, pharmacological activation of AMPK induced by compounds with mitochondrial action such as biguanides^70^ reverses mesenchymal phenotypes and inhibits metastasis in breast cancer^71–73^. Similarly, we describe that FR58P1a induces Sirt1/AMPK activation-dependent inhibition on TNBC cell migration, which is mediated by a decrease in cell surface presentation of β1-integrin. Likewise, FCCP activates the Sirt1/AMPK signaling; however, the anti-migratory effect is non-cancer cell selective. This difference may lie in the effect of FCCP at the plasma membrane, as it has been described that its depolarization generates both actin cytoskeletal and focal adhesion reorganization^74,75^. Cell migration is dependent on the dynamic formation and disassembly of actin filament-based structures, including lamellipodia, filopodia and invadopodia, as well as on cell-cell and cell-extracellular matrix adhesions^76^, all of which are modulated to some extent by one or more ionic species. Thus, depolarization of the plasma membrane generates an ion imbalance that reduces cell migration^77^. Therefore, the off- and on-target effects of FCCP may contribute to non-selective anti-migratory actions observed in this study.

FR58P1a produces an early fragmentation of the mitochondrial network characterized by formation of ring-like structures which have been associated with a metabolic stress^29^, an event that may be mediated by OXPHOS uncoupling-induced mitochondrial depolarization. It have been described that OXPHOS uncoupler-treated cells generate an adaptive response over prolonged periods which promote mitochondrial biogenesis and enhanced oxidative metabolism^47,48^. Conversely, prolonged FR58P1a treatment generates a metabolic adaptation toward glycolysis that includes an upregulation of the glucose transporter 4 (GLUT4), which is known to play an essential role in basal glucose uptake in breast cancer cells promoting proliferation and survival under hypoxic conditions^78^, a downregulation of OXPHOS-related genes (*cox-iv isoform 1*, *cyt c*, *atp5fa1*), reduced cardiolipin content, and reduced expression of respiratory complexes (II, IV, V) and proteins of outer mitochondrial membrane (VDAC, TOMM20). In glycolysis-dominant cancer cells, the mitochondrial respiration has an essential role in providing access to electron acceptors, such as NAD^+^^42,79,80^. Consistent with this, ETC inhibitors (i.e. complex I inhibitors such as rotenone and metformin) affect proliferation by decreasing NAD^+^ levels and the NAD^+^/NADH ratio^81,82^. In contrast, uncouplers of OXPHOS lack the effect on proliferation because the NAD^+^ availability is not altered^42,79^. Accordingly, FR58P1a does not affect the cell cycle progression and proliferation of TNBC cells.

The Δψ_m_ is a key player in mitophagy, a fine quality control to remove damaged mitochondria via autophagy and promote mitochondrial biogenesis^83^. At 24 h, FR58P1a induces an increase in PINK1 levels, an initiator of mitophagy under metabolic stress that involves decreased Δψ_m_^50,51^. Although our preliminary observations suggest that FR58P1a generates a reduction in mitochondrial mass, a role for mitophagy in this phenomenon with implications in the inhibitory effect on migration of TNBC cells requires further studies. Recently a new anti-cancer small molecule that induces mitophagy through Sirt1/PINK1/Parkin pathway has shown to induce cell death in glioblastoma *in vivo*^84^.

Here, we identified FR58P1a, a new mild OXPHOS uncoupler that actives the Sirt1/AMPK axis and triggers a metabolic adaptation toward glycolysis, that promotes cancer cell survival but selectively decreases the migratory capability of TNBC cells. The design of small molecules with mitochondrial action may offer a solution to the unresolved problem of metastasis generation.

## Material and Methods

### Reagents

All reagents were obtained from Sigma-Aldrich Corp. (St. Louis, MO, USA). Stock solutions of all compounds were prepared in dimethyl sulfoxide (DMSO).

### Chemicals

Duroquinol was synthesized by reduction from duroquinone as previously described^85^. The compounds FR58H8-11, FR58C1-4 and FR58BF1-BF6 have been previously reported by us^86–92^ and new compounds FR58P1-6a/b were obtained according to the Scheme 1 showed in the Supplementary Information, Experimental Section. Details on the reagents, apparatus and general procedures used to obtain the compounds and spectra analysis are shown in Supplementary Information.

### Cell lines

Mouse mammary adenocarcinoma TA3/Ha cell line was kindly provided by Dr. Gabriel Jose Gasic, UPENN, being used by our laboratory since 1989^93^. Human triple-negative breast cancer MDA-MB-231, MDA-MB-468 and BT-549 cell lines were purchased from American Tissue Culture Collection (ATCC).

### Cell culture conditions for screening of compounds with mitochondrial actions

Breast cancer MDA-MB-231 cells were grown in Dulbecco’s modified Eagle’s medium (DMEM), containing 25 mM glucose and 4 mM glutamine (+glu/+gln) supplemented with 10% fetal bovine serum (FBS), penicillin (100 IU/mL) and streptomycin (100 µg/mL). For the generation of cellular subpopulations with different metabolic phenotypes, a fraction of MDA-MB-231 cells were maintained in the same media described above without glucose and with 10 mM galactose (−glu/+gln) instead, for 7 days to adapt their metabolic profiles. All culture media contained no exogenous pyruvate supplementation. The metabolic remodeling from glycolysis to OXPHOS was confirmed by evaluating intracellular ATP levels after 2 h exposure to oligomycin. All cells were maintained in a humidified atmosphere at 37 °C and 5% CO_2_.

### MTT assay and S-value determination

The MTT assay was used to evaluate cellular proliferation as described previously^55^. Briefly, 1 × 10^4^ cells/100 µL were seed in 96-well microtiter plates and incubated in either +glu/+gln or −glu/+gln media for 24 h. The cells were then treated during 48 h with increasing concentrations of *ortho*-carbonyl compounds, mitochondriotoxic and glycolytic drugs to obtain a dose-response curve and obtaining the IC_50_ values in both conditions. To identify compounds with mitochondrial action, a nutrient-sensitized index (S-value) was calculated; log(IC_50_ in complete medium/IC_50_ in galactose medium) as described^25^. In this work, we considered a compound with a relevant and specific mechanism in a metabolic pathway when S-value was > +1 (mitochondrial metabolism-affecting compounds) or < −1 (glycolysis-affecting compounds). The quality of the small molecules screening was assessing the Z´factor according to reported by Zhang *et al*.^94^.

### Cellular respiration in real time

Multiparameter metabolic analysis of MDA-MB-231 cells was performed in an extracellular flux analyzer XFe96 (Seahorse Bioscience, USA) as described^95^. Details are provided in the supplementary document.

### Respiration of isolated tumor mitochondria

Mitochondria were isolated from TA3/Ha ascites tumor cell line by fractional centrifugation as previously described^26^. Mitochondrial respiration was measured polarographically at 25 °C with a Clark electrode No. 5331 as described^55^. Details are provided in the supplementary document.

### Determination of intracellular ATP, NAD(P)H, ROS and mitochondrial membrane potential (∆Ψ_m_)

ATP levels were determined with CellTiter-Glo Luminescent Cell Viability Assay kit (Promega, USA) according to the manufacturer’s specifications. Intracellular NAD(P)H levels were measured by auto-fluorescence using specific excitation and emission wavelengths of 340/428 nm as described^26^. Mitochondrial membrane potential (∆Ψm) and intracellular ROS levels in intact cells were determined by flow cytometry using the potentiometric probe tetramethylrhodamine methyl ester (TMRM, Molecular Probe) and dihydroethidium (DHE) probe, respectively. Details are provided in the supplementary document.

### Viability assay, cell cycle analysis and cell count

The viability and cell cycle analysis were determined by flow cytometry and cell number was counted by trypan blue exclusion as is detailed in supplementary document.

### Western blotting

Details of Western blotting are available in supplementary document.

### Cell morphology assay

MDA-MB-231 cells were exposed to DMSO (Control) or FR58P1a (30 µM) for 4 h. Cells were then resuspended and seeded in culture medium without FBS in a 24-wells plate coated with fibronectin for 2 h. After stimulation, the cells were fixed with 4% paraformaldehyde, permeabilized using 0.1% Triton x-100 and stained with phalloidin. The cells were imaged using Leica DFC450 fluorescence microscope.

### Migration and adhesion assays

Cell migration was evaluated in Boyden Chamber assays (Transwell Costar, 6.5-mm diameter, 8-µm pore size) according to manufacturer’s instructions. Briefly, bottom sides of inserts were coated with fibronectin (2 µg/ml) and re-suspended TNBC cells in serum free medium were plated on top of the chamber insert and incubated at 37 °C for 2 h. Then, inserts were removed, washed and the bottom side of the inserts stained with 0.1% crystal violet in 2% ethanol. Cells from eight different frames were counted for each condition in an inverted microscope. To evaluate cellular adhesion, MDA-MB-231 cells were exposed to DMSO or FR58P1a (10 and 30 µM) for 4 h and seeded (1 × 10^5^ cells/ml) in 96-well plates coated with fibronectin (2 µg/ml) or 0.01% poly-lysine followed by 1 h of incubation at 37 °C (5% CO_2_). Then, cells were fixed with 3% paraformaldehyde (10 min), washed with 2% methanol (10 min) and stained with 0.5% crystal violet (10 min). Finally, absorbance was measured at 540 nm.

### Quantification of β1-integrin on the cell surface

MDA-MB-231 cells were incubated for 4 h with DMSO, 30 µM FR58P1a, 10 µM Compound C (CC) or the combination CC + FR58P1a. Then, cells were resuspended and seeded in culture medium without FBS in a 6-wells plate coated with fibronectin (2 µg/ml) for 2 h. Cells were then collected and incubated at 4 °C to avoid internalization of surface proteins, inmunolabeled with primary antibody anti-β1 integrin (1:25) and secondary antibody anti-rabbit Alexa 488 (1:200) according to Rosfjord and Dickson^96^. The presence of β1-integrin on the cell surface was detected by flow cytometry.

### Determination of cardiolipin content and 2NBDG uptake

Details are available in supplementary document.

### Electrophysiological recordings and live-cell confocal microscopy

Details are available in supplementary document.

### RNA isolation, cDNA synthesis and qPCR

Details are available in supplementary document.

### Statistics

All statistical analyses were performed using Graph Pad Prism 4.03 (GraphPad Software, San Diego, California, USA). The data are expressed as mean ± SEM of three independent experiments, each one performed in technical triplicate. Statistical analysis was performed using one-way or two-way ANOVA with Bonferroni’s post-test for pairwise comparisons. The data were considered statistically significant when p < 0.05.

## Electronic supplementary material

Supplementary Information

## Data Availability

The authors declare that all the materials, data and associated protocols related to this manuscript are available without conditions.
