# Supplementary material for: FR58P1a; a new uncoupler of OXPHOS that inhibits migration in triple-negative breast cancer cells via Sirt1/AMPK/β1-integrin pathway
Source: Sci Rep. 2018 Sep 4;8:13190. doi: 10.1038/s41598-018-31367-9 (PMC6123471; doi:10.1038/s41598-018-31367-9)
Supplement: Supplementary file 1 — Supplementary Information [file 41598_2018_31367_MOESM1_ESM.docx]

**FR58P1a; a new uncoupler of OXPHOS that inhibits migration in triple-negative breast cancer cells via Sirt1/AMPK/β1-integrin pathway**

Félix A. Urra, Felipe Muñoz, Miguel Córdova-Delgado, María Paz Ramírez, Bárbara Peña-Ahumada, Melany Rios, Pablo Cruz, Ulises Ahumada-Castro, Galdo Bustos, Eduardo Silva-Pavez, Rodrigo Pulgar, Danna Morales, Diego Varela, Juan Pablo Millas-Vargas, Evelyn Retamal, Oney Ramírez-Rodríguez, Hernán Pessoa-Mahana, Mario Pavani, Jorge Ferreira, César Cárdenas and Ramiro Araya-Maturana

**Supplementary Information**

**Extended Material and Methods**

**Antibodies and reagents.** All reagents were obtained from Sigma-Aldrich Corp. (St. Louis, MO, USA). Rabbit antibody anti-β1 integrin (#SC9878, Santa Cruz Biotechnology), rabbit antibody phospho-AMPKα Thr172 (#2531, Cell Signaling Technology), rabbit antibody total AMPKα (#2532, Cell Signaling Technology), rabbit antibody VDAC (#AB10527, Merck Millipore), rabbit antibody TOM20 (#42406S, Cell Signaling Technology), Total OXPHOS mouse antibody cocktail (#MS601, Mitosiences Inc), mouse anti-β-tubulin (#322600, Invitrogen), Alexa Fluor-488 phalloidin (#A12379, Invitrogen) and secondary rabbit antibody Alexa 488 (#A11034, Invitrogen) were used as indicated by the manufacturers.

**Cellular respiration in real time.** Oxidative and glycolytic subpopulations of breast cancer MDA-MB-231 (20.000 cells/well) were seeded on XFe96 V3-PS multi-well plates, and kept overnight at 37 °C in 5 % CO_2_ with culture medium containing galactose plus glutamine or glucose plus glutamine, respectively. The next day, the culture medium was replaced with assay media (unbuffered DMEM without red phenol, with 4 mM glutamine and 10 mM galactose, pH = 7.4 or 4 mM glutamine and 10 mM glucose, pH = 7.4) 1 h before the assay. Mitochondrial function was evaluated using 1 µM oligomycin, 100 nM FCCP, 1 µM rotenone and 1 µM antimycin-A. Oxygen consumption rate (OCR) and extracellular acidification rate (ECAR) measurements were made with specific excitation and emission wavelengths of oxygen (532/650 nm) and protons (470/530 nm). Each experiment was run in triplicate.

**Respiration of isolated tumor mitochondria.** To determine the effect of selected compounds on the OXPHOS, mitochondria were stimulated with substrates for respiratory chain complex I (4.2 mM glutamate + 4.2 mM malate), complex II (5.0 mM succinate + 0.170 µM rotenone), complex III (0.30 mM duroquinol) and complex IV (1 µM antimycin + 0.075 mM TMPD + 1.5 mM ascorbate) in presence of ADP (0.25 mM), reaching state 3_ADP_. To evaluate the effect of the compounds FR58P1a and H8 on state 3u (CCCP-stimulated respiration) and state 4o (proton leak-driven respiration), 200 nM CCCP and 2 µM oligomycin were added, respectively. The Respiratory Control Ratio (RCR) was calculated as the respiration in state 3_ADP_ divided by that in state 4. To evaluate the participation of mPTP and ANT in the uncoupling effect induced by FR58P1a, 1 µM cyclosporine A (CsA, with 2 min of incubation) and 30 µM atractyloside (Atrac) were used, respectively. To reverse the protonophoric effect of hydroquinones, 100 µM 6-ketocholestanol was used. K_0.5_-values were obtained from double-log regression as described^1^.

**Determination of intracellular ATP, NAD(P)H and mitochondrial membrane potential (∆Ψ_m_).** ATP levels were determined with CellTiter-Glo Luminescent Cell Viability Assay kit (Promega, USA) according to the manufacturer´s specifications. MDA-MB-231 cells (1x10^5^ cells/mL) were seeded into 96-well plates, incubating for 1 and 4 h in culture medium in the absence (control in DMSO) or presence of FR58P1a. After exposure, the cells were washed twice with PBS to remove the culture medium and re-suspended in 20 μL PBS. The bioluminescence was measured as described previously^2^.

Intracellular NAD(P)H levels were measured by auto-fluorescence using specific excitation and emission wavelengths of 340/428 nm^3^. TNBC cells were collected and resuspended in 1 mL of HBSS in a cuvette. Consecutive injections of FCCP or FR58P1a and rotenone were used to obtain the mitochondrial NADH oxidation as described^4^.

The generation of intracellular oxidative stress was determined using the dihydroethidium (DHE) probe. TNBC cells were grown in complete media, seeded in 12-well plates allowed overnight to attach. Then, cells were exposed for 1 or 4 h to DMSO (Control) or 30 µM FR58P1a, at the end of the exposure time, culture media was replaced by a solution containing 5 µM DHE in Hank's balanced salt solution (HBSS) and incubated for 20 min in the dark. After this time, cells were washed, trypsinized, and resuspended in 200 µL of HBSS and measured by FACS Calibur flow cytometer.

Mitochondrial membrane potential (∆Ψm) in intact cells was determined by flow cytometry using the potentiometric probe tetramethylrhodamine methyl ester (TMRM, Molecular Probe). Briefly, suspensions of MDA-MB-231 (1.5 x 10^5^ cells/mL) were treated with DMSO (Control) or 30 µM FR58P1a for 4 h. FCCP (200 nM) was used as positive control. The cells were then washed with PBS and incubated with 5 nM TMRM for 20 min. Cells were collected, washed, re-suspended and fluorescence was detected using a FACS Calibur flow cytometer.

**Cell viability assay, cell cycle analysis and cell count.** The cell viability was evaluated using the ability of cells to exclude propidium iodide (PI). In brief, TNBC cells were collected from 24-well plates after exposition with DMSO (Control) or 10 and 30 µM FR58P1a for 48 h and the level of PI incorporation was quantified in a FACScan flow cytometer (Becton-Dickinson, San Jose, CA). PI-negative subpopulations of TNBC cells were considered viable. To estimate cell cycle distribution, cellular DNA contents were measured by flow cytometry as previously described^2^. TNBC cells were incubated with DMSO (Control) or 10 and 30 µM FR58P1a for 48 h. All samples were analyzed for cell cycle distribution using a FACS Calibur flow cytometer and the Becton–Dickinson Cell Quest Acquisition software (San Jose, CA, USA). Data were reported as percentage of cells in each phase of the cell cycle. To evaluate cell number, MDA-MB-231 cells (25,000 cells) were seeded into 24-well plates and incubated for 24 h. Then, the cells were exposed to DMSO (Control) or FR58P1a until 72 h. After treatment, MDA-MB-231 cells were counted by staining with 0.4 % solution of trypan blue, using a hemacytometer and optic microscopy as described ^3^.

**Western blotting.** TNBC cells were exposed to DMSO (Control) or FR58P1a (10 and 30 µM) for 4 h, and lysed on ice with Cytobuster protein extraction reagent (Novagen) supplemented with protease and phosphatase inhibitors (complete PhosSTOP, Roche). The protein quantification was performed by Bradford assay. Protein extracts were separated in 10 % SDS-polyacrylamide gels and transferred to PDVF membranes (Millipore). They were blocked in 5 % fat-free milk for 1 h at room temperature and incubated overnight at 4 °C with primary antibody followed by incubation for 1 h at room temperature with a secondary antibody conjugated to horseradish peroxidase. Chemiluminescence detection and densitometric analyses were done with ImageJ software as described^5^.

**Determination of cardiolipin content and 2NBDG uptake.** Cardiolipin content was measured using acridine orange 10-nonyl bromide (NAO, Invitrogen)^6^. Cells were seeded in 12-well plates overnight. Cells were treated with DMSO (Control) or 30 µM FR58P1a for 24 h. Then, MDA-MB-231 cells were washed in cold-HPBS and incubated with 100 nM NAO in HBSS at 37 °C for 20 min in the dark. After this time, cells were washed, collected and resuspended in 200 µl of HBBS. NAO fluorescence was measured by flow cytometry. To measure glucose uptake, the fluorescent substrate 2-(N-(7-nitrobenz-2-oxa-1,3-diazol-4-yl)amino)-2-deoxyglucose (2-NBDG) was used^7^. Cells treated with DMSO (Control) or 30 µM FR58P1a for 4 and 24 h. Then, fresh DMEM Low glucose medium containing 100 µM 2-NBDG was added and cells were incubated during 30 min at 37 °C in the dark. After this time, cell were washed, collected and resuspended in 200 µl of cold HBBS. The 2-NBDG fluorescence was measured by flow cytometry.

**Live-cell confocal microscopy.** MCF-10A and BT-549 cells grown on 25 mm glass coverslips were incubated for 4 h with DMSO (control), 30 μM FR58P1a and 1 μM FCCP and then incubated 30 min with 1 μM MitoTracker^®^ Green FM (Thermo Fisher Scientific). Images were acquired through 63 X oil immersion objective with a Nikon C2 plus confocal microscope. ﻿Confocal 8-bit RGB images taken were processed with ImageJ (National Institutes of Health).

**Electrophysiological recordings.** Membrane potential of MDA-MB-231 breast cancer cells was recorded at room temperature (18-21°C) using Patch-Clamp in whole-cell recording mode. Borosilicate glass pipettes (World Precision Instrument, Sarasota, FL) were pulled to have 2–4-MΩ resistance using a P70 horizontal puller (Sutter Instruments) and filled with internal solution containing 105 mM K-Glu, 35 mM KCl, 8.8 mM NaCl, 10 mM HEPES, 0.5 mM EGTA, 30 mM Sucrose, 0.4 mM GTP-Na salt, 5 mM ATP-Na salt (pH 7.2 adjusted with KOH); the bath solution contained 140 mM NaCl, 4 mM KCl, 2 mM CaCl_2_, 1 mM MgCl_2_, 10 mM HEPES, 10 mM glucose (pH 7.4 adjusted with NaOH). Membrane potentials were recorded in current clamp with a gap free protocol, using a 1550b Digidata (Axon Instruments) and an EPC7 amplifier (HEKA Elektronik). Data acquisition and analysis were performed using Clampfit 9.2 (Axon Instruments).

**RNA isolation.** Three biological replicates of total RNA were extracted independently from 1x10^6^ MDA-MB-231 cell treated with FR58P1a (30 µM) by 24 and 72 h using TRIZOL Reagent (Invitrogen) and were purified using RNeasy mini kit (Qiagen) according to the manufacturer’s instructions. The quantity of the total RNA was determined using a Qubit Fluorometric Quantitation System (Life Technologies) and the purity (absorbance 260/280 nm) using a NanoQuant Spectrophotometer (Tecan Technologies), while integrity was confirmed by RNA Integrity Number (RIN) using a 2200 TapeStation Instrument (Agilent Technologies, Inc). Only samples of high quality (Absorbance 260/280 nm ≥ 1.9 and RIN ≥ 8.5) were used for gene expression analyses.

**cDNA synthesis and qPCR.** Two μg of total RNA was used as template for reverse transcription reactions to synthesize cDNA using High-Capacity RNA to cDNA Kit (Thermo Fisher Scientific), according to standard procedures. cDNAs were diluted to 100 ng and used as templates for qPCR, reactions that were carried out on a AriaMx real-time PCR system (Agilent Technologies) using Terra qPCR Direct TB Green Premix (Takara). PCR conditions were 95 °C for 5 min followed by 94 °C for 15 s, 58–64 °C for 15 s and 72 °C for 20 s for a total of 35 cycles. Melting curves (1 °C steps between 75–95 °C) ensured that a single product was amplified in each reaction. To determine relative expression levels of the genes, the method described by Pfaffl^8^ and adapted by Talke and coworkers^9^, was used. Human Glyceraldehyde-3-phosphate dehydrogenase was selected as the internal reference gene. At least three biological replicates and three technical replicates were performed and differences among conditions were analyzed using Student’s t-test (p < 0.05) or One-way ANOVA. PCR efficiencies were determined by linear regression analysis performed directly on the sample data using LinRegPCR^10^. Supplementary Table 2 shows the complete list of primers used in this study.

**EXPERIMENTAL SECTION: Synthesis of *ortho*-carbonyl substituted scaffold-containing compounds.**

The compounds FR58H8-11, FR58C1-4 and FR58BF1-BF6 have been previously reported by us ^11-17^ and new compounds FR58P1-6a/b were obtained according to the Scheme 1.

**Supplementary Scheme 1: Synthesis of *ortho*-carbonyl substituted hydroquinones and benzofuranes.**

**Chemicals, reagents, general procedures and apparatus.** ^1^H and ^13^C NMR spectra were obtained from a spectrometer operating at either 300.13 MHz (^1^H) or 75.47 MHz (^13^C). Measurements were carried out at 300 K in CDCl_3_. Chemical shifts are reported as ppm downfield from TMS for ^1^H NMR and relative to the central CDCl_3_ resonance (77.0 ppm) for ^13^C NMR. All melting points are uncorrected and were determined using an Electrothermal 9100 apparatus. IR spectra (KBr discs) were recorded on a FT-IR spectrophotometer; wave numbers are reported in cm^-1^. High resolution mass spectra were obtained on an orthogonal time-of-flight (Tof) mass spectrometer (QTof Micro, Micromass UK) or on a Thermo Q exactive focus. Silica gel 60 (230-400 mesh ASTM) and TLC aluminium sheets silica gel 60 F254 were used for flash-column chromatography and analytical TLC, respectively.

**Procedure to synthesize acylhydroquinones.** The corresponding hydroquinone (**FR58H1** or **FR58H2**), the carboxylic acid (1.5 eq.) and BF_3_^.^2H_2_O (4 mL), were mixed in a sealed glass tube for microwave reactor, equipped with a magnetic bar. The mixture was stirred and irradiated at 120 °C and 150 W for 20 minutes. Then was diluted with water and extracted with ethyl acetate. The organic phase was washed with water, dried with magnesium sulfate and concentrated in vacuum. The crude was purified by column flash chromatography on silica gel, using petroleum ether /EtOAc (6:1).

**Synthesis of the tricyclic hydroquinone derivatives.** 8,8-dimethyl-1,4,5(8*H*)-naphthalenetrione (**Q1**)^12^, and the corresponding (2*E*,4*E*)-2,4-hexadien-1-yl ester were dissolved in 5 mL of toluene in a round-bottomed flask for a week, then silica gel was added to the reaction mixture and stirred overnight. The regioisomers, were purified by flash column chromatography. A key entry for the assignment, in this series of hydroquinone derivatives, is the presence of the chelated hydroxyl group at C-10. The corresponding proton, resonating at about 13 ppm, show *J*_C,H_ correlations, in HMBC-spectra with C-4a and C-10a, as C-4a also correlates with methyl group at C-4 these correlations allow the regiochemical assignations.

***(9,10-dihydroxy-4,8,8-trimethyl-5-oxo-1,4,5,8-tetrahydroanthracen-1-yl)methyl heptanoate (FR58P1a)*** and ***(9,10-dihydroxy-4,5,5-trimethyl-8-oxo-1,4,5,8-tetrahydroanthracen-1-yl)methyl heptanoate (FR58P1b)*.** 112 mg (0.55 mmol) of Q1, react with 153 mg (0.55 mmol) of (2*E*,4*E*)-2,4-hexadien-1-yl 6-bromohexanoate in 10 mL of toluene. The regioisomers were purified by column chromatography using as eluent a mixture hexane: dichloromethane: ethyl acetate (20:20:1). ***FR58P1a*** (90 mg, 33 % yield), ^1^H-NMR δ(CDCl_3_): 1.39 (d, *J* = 7.0 Hz, 3H, 4-CH_3_), 1.46 (m, 2H, CH_2_), 1.59 (s, 3H, 5-CH_3_), 1.65 (s, 3H, 5-CH_3_), 1.65 (m, 2H, CH_2_), 1.85 (q, *J* = 6.8 Hz, *J* = 7.4 Hz, 2H, CH_2_), 2.31 (t, *J* = 7.5 Hz, 2H, CH_2_), 3.39 (t, *J* = 6.8 Hz, 2H, CH_2_), 3.49 (m, 1H, CH), 4.03 (m, 1H, CH), 4.19 (dd, *J* = 7.3 Hz, *J* = 10.2 Hz, 1H, CHH-O), 4.48 (dd, *J* = 4.1 Hz, *J* = 10.2 Hz, 1H, CHH-O), 4.69 (*s*, 1H, 10-OH), 6.00 (dd, *J* = 4.1 Hz, *J* = 9.9 Hz, 1H, 2- or 3-H), 6.05 (dd, *J* = 4.3 Hz, *J* = 9.9 Hz, 1H, 2- or 3-H), 6.24 (d, *J* = 10.1 Hz, 1H, 7-H), 6.83 (d, *J* = 10.1 Hz, 1H, 6-H), 13.27 (s, 1H, 9-OH). ^13^C-NMR δ(CDCl_3_): 22.41, 23.96, 25.07, 25.25, 27.61, 30.19, 32.35, 33.47, 33.95, 34.36, 38.12, 67.20, 112.9, 121.4, 123.9, 126.2, 131.2, 133.3, 136.7, 142.6, 154.7, 161.0, 173.5, 191.1. M.p. 99–100.5 ºC, IR ν(cm^-1^) 1733, 2952, 3047, 3422 HRMS (ESI) *m/z* M^+^ Calcd. for C_24_H_29_BrO_5_: 476.11984 found: 476.11942. ***FR58P1b*** (170 mg, 63 % yield), ^1^H-NMR δ(CDCl_3_): 1.33 (d, *J* = 7.0 Hz, 3H, 4-CH_3_), 1.51 (m, 2H, CH_2_), 1.61 (*s*, 3H, 8-CH_3_), 1.68 (*s*, 3H, 8-CH_3_), 1.72 (q, *J* = 7.3 Hz, *J* = 7.9 Hz, 2H, CH_2_), 1.89 (q, *J* = 6.7 Hz, *J* = 7.4 Hz, 2H, CH_2_), 2.45 (t, *J* = 7.3 Hz, 2H, CH_2_), 3.42 (t, *J* = 6.7 Hz, 2H, CH_2_), 3.75 (m, 1H, CH), 3.83 (m, 1H, CH), 3.95 (dd, *J* = 7.6 Hz, *J* = 10.8 Hz, 1H, CHH-O), 4.33 (dd, *J* = 5.3 Hz, *J* = 10.8 Hz, 1H, CHH-O), 5.94 (dd, *J* = 4.9 Hz, *J* = 9.9 Hz, 1H, 2- or 3-H), 6.17 (dd, *J* = 5.0 Hz, *J* = 9.9 Hz, 1H, 2- or 3-H), 6.24 (d, *J* = 10.1 Hz, 1H, 6-H), 6.85 (d, *J* = 10.1 Hz, 1H, 7-H), 6.89 (s, 1H, 9-OH), 13.18 (s, 1H,10-OH). ^13^C-NMR δ(CDCl_3_): 22.38, 23.83, 24.91, 25.15, 27.51, 29.59, 32.21, 33.26, 33.92, 35.21, 38.32, 71.00, 113.7, 123.2, 123.9, 127.8, 130.0, 132.8, 134.6, 143.9, 153.9, 161.4, 174.5, 191.4. M.p.108.2-108.9 ºC; IR ν(cm^-1^): 1710, 2963, 3033, 3336 . HRMS (ESI) *m/z* M^+^ Calcd. for C_24_H_29_BrO_5_: 476.11984 found: 476.11926.

***(9,10-dihydroxy-4,8,8-trimethyl-5-oxo-1,4,5,8-tetrahydroanthracen-1-yl)methyl heptanoate (FR58P2a)*** *and* ***(9,10-dihydroxy-4,5,5-trimethyl-8-oxo-1,4,5,8-tetrahydroanthracen-1-yl)methyl heptanoate (FR58P2b).*** 373 mg (1.78 mmol) of Q1, react with 405 mg (1.78 mmol) of (2*E*,4*E*)-2,4-hexadien-1-yl heptanoate in 10 mL of toluene. The regioisomers were purified by column chromatography using as eluent a mixture hexane: ethyl acetate: acetone (80:20:0.5). ***FR58P2a*** (319 mg, 43 % yield), ^1^H NMR δ(CDCl_3_): 0.90(t, *J* = 6.9 Hz, 3H, CH_3_); 1.25-1.42(m, 8H, 4 x CH_2_); 1.34(d, *J* = 7 Hz, 3H, CH_3_); 1.62(s, 3H, CH_3_); 1.63-1.75(m, 2H, CH_2_); 1.68(s, 3H, CH_3_); 2.42(t, *J* = 7.7 Hz, 2H, CH_2_); 3.71-3.87(m, 2H, 2 x CH); 3.96(dd, *J*_1_ = 7.5 Hz, *J*_2_ = 10.8 Hz, 1H, CHH-O); 4.33(dd, *J*_1_ = 5.4 Hz, *J*_2_ = 10.8 Hz, 1H, CHH-O); 5.95(dd, *J*_1_ = 5Hz, *J*_2_ = 9.8 Hz, 1 H, 2- or 3-H); 6.20(dd, *J*_1_ = 5 Hz, *J*_2_ = 9.8 Hz, 1H, 2- or 3-H); 6.25(d, *J* = 10 Hz, 1H, 6-H); 6.85(d, *J* = 10 Hz, 1H, 7-H); 6.96(s, 1H, 9-OH); 13.18(s, 1H, 10-OH). ^13^C-NMR δ(CDCl_3_): 14.01; 22.45; 24.75; 24.95; 25.20; 28.75; 29.65; 31.38; 34.27; 35.50; 38.59; 70.98; 113.70; 123.28; 123.93; 127.87; 130.18; 132.82; 134.65; 143.99; 153.96; 161.47; 174.99; 191.45. IR ν(cm^-1^): 1705.63, 2961.25, 3035.45, 3397.87 HRMS (ESI): calcd. for C_25_H_32_O_5_ *m/z* M^+^: 412,22497, found: 412.22101. ***FR58P2b*** (324 mg, 44 % yield), ^1^H NMR δ(CDCl_3_): 0.87(t, *J* = 7 Hz, 3H, CH_3_); 1.25-1.34(m, 6H, 3 x CH_2_); 1.39(d, *J* = 7 Hz, 3H, CH_3_); 1.52-1.76(m, 4H, 2 X CH_2_); 1.59(s, 3H, CH_3_); 1.65(s, 3H, CH_3_); 2.29(t, *J* = 7.6 Hz, 2H, CH_2_); 3.43-3.55(m, 1H, CH); 3.98-4.08( m, 1H, CH); 4.18(dd, *J*_1_ = 7.4 Hz, *J*_2_ = 10.2 Hz, 1H, CHH-O); 4.48(dd, *J*_1_ = 5.4 Hz, *J*_2_ = 10.2 Hz, 1H, CHH-O); 4.75(s, 1H, 10 OH); 6.00(dd, *J*_1_ = 4 Hz, *J*_2_ = 9.9 Hz, 1 H, 2- or 3-H); 6.04(dd, *J*_1_ = 4 Hz, *J*_2_ = 9.9 Hz, 1H, 2- or 3-H); 6.24(d, *J* = 10 Hz, 1H, 7-H); 6.82(d, *J* = 10 Hz, 1H, 6-H); 13.27(s, 1H, 10-OH). ^13^C-NMR δ(CDCl_3_): 13.98; 22.41; 24.80; 24.99; 25.19; 28.75; 30.16; 31.39; 34.25; 34.33; 38.14; 67.11; 112.88; 121.34; 123.87; 126.16; 131.12; 133.33; 136.90; 142.66; 154.64; 161.07; 174.03; 191.16. IR ν(cm^-1^): 1728.30, 2963.99, 3036.33, 3418.33 *m/z* HRMS (ESI): calcd. for C_25_H_32_O_5_ M^+^: 412,22497, found: 412.22092.

***(9,10-dihydroxy-4,8,8-trimethyl-5-oxo-1,4,5,8-tetrahydroanthracen-1-yl)methyl 5-phenylpentano-ate (FR58P3a)*** *and* ***(9,10-dihydroxy-4,5,5-trimethyl-8-oxo-1,4,5,8-tetrahydroanthracen-1-yl)methyl 5-phenylpentanoate (FR58P3b).*** 226 mg (1.12 mmol) of Q1, react with 289 mg (1.12 mmol) of (2*E*,4*E*)-2,4-hexadien-1-yl 5-phenylpentanoate in 10 mL of toluene yielding: ***FR58P3a*** (164.5 mg, 32 % yield), ^1^H-NMR δ(CDCl_3_):1.34(d, *J* = 6.9 Hz, 3H, 4-CH_3_); 1.62(s, 3H, CH_3_); 1.68(s, 3H, 8-CH_3_); 1.65-1.80(m, 4H, 2X CH_2_); 2.45(t, *J* = 7 Hz, 2H, CH_2_); 2.66(t, *J* = 6.7 Hz, 2H, CH_2_); 3.70-3.86(m, 2H, 2XCH); 3.94(dd, *J*_1_ = 7.8 Hz, *J*_2_ = 10.8 Hz, 1H, CHH-O); 4.32(dd, *J*_1_ = 5.4 Hz, *J*_2_ = 10.8 Hz, 1H, CHH-O); 5.93(dd, *J*_1_ = 4.9 Hz, *J*_2_ = 9.8Hz, 1H, 2- or 3-H); 6.18(dd, *J*_1_ = 5 Hz, *J*_2_ = 9.7 Hz, 2- or 3-H); 6.25(d, *J* = 10 Hz, 1H, 6-H); 6.85(d, *J* = 10 Hz, 3-H); 6.90(s, 1H, OH); 7.14-7.22(m, 3H, Ar.H); 7.24-7.33(m, 2H, Ar.H); 13.19(s, OH). ^13^C-NMR δ(CDCl_3_): 22.42; 24.33; 24.93; 25.19; 29.63; 30.77; 34.09; 35.25; 35.46; 38.36; 71.02; 113.69; 123.20; 123.91; 125.89; 127.86; 128.38; 129.37; 130.06; 132.79; 134.66; 141.77; 143.93; 153.94; 161.41; 174.71; 191.43. IR ν (cm^-1^): 1717.04, 2961.25, 3021.18, 3392.16 HRMS (ESI): calcd. for C_29_H_32_O_5_ *m/z* M^+^: 460.22497, found: 460.22052. ***FR58P3b*** (184 mg, 36 % yield), ^1^H-NMR δ(CDCl_3_):1.38(d, *J* = 7.1 Hz, 3H, 4-CH_3_); 1.60(s, 3H, 5-CH_3_); 1.62-1.69(m, 4H, 2X CH_2_); 1.65(s, 3H, 5-CH_3_); 2.28-2.36(m, 2H, CH_2_); 2.57-2.66(m, 2H, CH_2_); 3.42-3.58(m, 1H, CH); 3.99-4.07(m, 1H, CH); 4.18(dd, *J*_1_ = 7.5 Hz, *J*_2_ = 10.2 Hz, 1H, CHH-O); 4.49(dd, *J*_1_ = 4.1 Hz, *J*_2_ = 10.2 Hz, 1H, CHH-O); 4.66(bs, 1H, OH); 5.99(dd, *J*_1_ = 4 Hz, *J*_2_ = 9.9Hz, 1H, 2- or 3-H); 6.03(dd, *J*_1_ = 4 Hz, *J*_2_ = 9.9 Hz, 2- or 3-H); 6.24(d, *J* = 10 Hz, 1H, 7-H); 6.82(d, *J* = 10 Hz, 6-H); 7.14-7.21(m, 3H, Ar.H); 7.24-7.31(m, 2H, Ar.H); 13.28(s, 1H, 9-OH). ^13^C-NMR δ(CDCl_3_): 22.38; 24.45; 25.02; 25.22; 29.63; 30.16; 30.86; 34.06; 34.34; 35.53; 38.12; 67.12; 113.00; 121.44; 124.01; 125.89; 125.78; 128.38; 126.30; 128.32; 128.41; 131.14; 133.29; 136.71; 142.17; 142.65; 154.75; 161.06; 173.74; 191.21. IR ν(cm^-1^): 1734.08, 2966.88, 3024.76, 3392.28 HRMS (ESI): calcd. for C_29_H_32_O_5_ *m/z* M^+^: 460.22497, found: 460.22101.

***(9,10-dihydroxy-4,8,8-trimethyl-5-oxo-1,4,5,8-tetrahydroanthracen-1-yl)methyl 3-(4 chloro phenyl) propanoate (FR58P4a)*** and ***(9,10-dihydroxy-4,5,5-trimethyl-8-oxo-1,4,5,8-tetrahydroanthracen-1-yl)methyl 3-(4-chlorophenyl) propanoate (FR58P4b)***

305 mg (1.51 mmol) of 8,8-dimethyl-1,4,5(8H)-naphthalenetrione^18^ react with 400 mg (1.51 mmol) of (2*E*,4*E*)-2,4-hexadien-1-yl 3-(4-chlorophenyl)propanoate in 10 mL of toluene yielding: ***FR58P4a***,(253 mg, 36 % yield), ^1^H-NMR δ(CDCl_3_):1.33(d, *J* = 6.9 Hz, 3H, 8-CH_3_); 1.61(s, 3H, CH_3_); 1.67(s, 3H, CH_3_); 2.73(t, *J* = 7.6 Hz, 2H, CH_2_); 2.99(t, *J* = 7.6 Hz, CH_2_); 3.69-3.81(m, 2H, 2XCH); 3.92(dd, *J*_1_ = 8 Hz, *J*_2_ = 10.5 Hz, 1H, CHH-O-); 4.33(dd, *J*_1_ = 5.2Hz, *J*_2_ = 10.8 Hz, 1H, CHH-O-); 5.83(dd, *J*_1_ = 4.7 Hz, *J*_2_ = 9.7Hz, 1H, 6- or 7-H); 6.16(dd, *J*_1_ = 4.8 Hz, *J*_2_ = 9.7 Hz, 6- or 7-H); 6.25(d, *J* = 10 Hz, 1H, 2-H); 6.79(s, 1H, 9-OH); 6.86(d, *J* = 10 Hz, 3-H); 7.15(d, *J* = 8.2 Hz, 2H, Ar-H); 7.28(d, *J* = 8 Hz, 2H), 13.19(s, 10-OH). ^13^C-NMR δ(CDCl_3_): 22.41; 24.98; 25.22; 29.65; 30.09; 35.21; 35.65; 38.37; 71.19; 113.73; 123.10; 123.97; 127.91; 128.80; 129.62; 129.89; 132.43; 132.78; 134.73; 138.21; 143.86; 154.00; 161.39; 173.86; 191.43. IR ν(cm^-1^): 1748.55, 2995.82, 3036.33, 3476.21. HRMS (ESI) calcd for C_27_H_26_ClO_5_ *m/z* [M-1]^+^: 465,14743, found 465.14755. ***FR58P4b***, (371 mg, 53 % yield), ^1^H-NMR (300 MHz, CDCl_3_) δ: 1.37(d, *J* = 7.1 Hz, 3H, 4-CH_3_); 1.60(s, 3H, 5-CH_3_); 1.65(s, 3H, 5-CH_3_); 2.59(t, *J* = 7.4 Hz, 2H, CH_2_); 2.90(t, *J* = 7.7 Hz, 2H, CH_2_); 3.41-3.52(m, 1H, CH); 3.97-4.05(m, 1H, CH), 4.18(dd, *J*_1_ = 7.2 Hz, *J*_2_ = 10.3 Hz, 1H, CHH-O-); 4.47(dd, *J*_1_ = 4.1Hz, *J*_2_ = 10.2 Hz, 1H, CHH-O-); 4.58(s, 1H, 10-OH); 5.92(dd, *J*_1_ = 4.6 Hz, *J*_2_ = 9.9Hz, 1H, 2- or 3-H); 6.02(dd, *J*_1_ = 4.8 Hz, *J*_2_ = 9.9 Hz, 1H, 2- or 3-H); 6.26(d, *J* = 10.1 Hz, 1H, 7-H); 6.84(d, *J* = 10.1 Hz, 1H, 6-H); 7.10(d, *J* = 8.4 Hz, 2H, Ar-H); 7.24(d, *J* = 8.4 Hz, 2H, Ar-H), 13.28(s, 9-OH). ^13^C-NMR δ(CDCl_3_): 22.38; 25.02; 25.21; 29.65; 30.14; 34.25; 35.71; 38.13; 67.32; 112.93; 121.26; 123.92; 126.05; 128.52; 129.66; 131.14; 131.92; 133.28; 136.11; 138.91; 142.58; 154.65; 161.03; 172.65; 191.14. IR ν(cm^-1^): 1742.77, 2998.71, 3045.02, 3374.92. HRMS (ESI) calcd. for C_27_H_27_ClO_5_ *m/z* M^+^ : 466,15470, found 466.15063.

***(9,10-dihydroxy-4,8,8-trimethyl-5-oxo-1,4,5,8-tetrahydroanthracen-1-yl)methyl 3-(2-fluoro phenyl) propanoate (FR58P5a) and (9,10-dihydroxy-4,5,5-trimethyl-8-oxo-1,4,5,8-tetrahydroanthracen-1-yl)methyl 3-(2-fluorophenyl) propanoate (FR58P5b).*** 282 mg (1.40 mmol) of Q1, react with 357 mg (1.44 mmol) of (2*E*,4*E*)-2,4-hexadien-1-yl 3-(-fluorophenyl)propanoate in 10 mL of toluene yielding: ***FR58P5a*** (253 mg, 39 % yield), ^1^H-NMR δ(CDCl_3_):1.32(d, *J* = 6.9 Hz, 3H, 4-CH_3_); 1.61(s, 3H, 8-CH_3_); 1.67(s, 3H, 8-CH_3_); 2.75(t, *J* = 7.6 Hz, 2H, CH_2_); 3.01(t, *J* = 7.6 Hz, 2H, CH_2_); 3.69-3.81(m, 2H, 2 x CH); 3.94(dd, *J*_1_ = 7.8 Hz, *J*_2_ = 10.2 Hz, 1H, CHH-O); 4.34(dd, *J*_1_ = 5.2 Hz, *J*_2_ = 10.9 Hz, 1H, CHH-O); 5.84(dd, *J*_1_ = 4.7 Hz, *J*_2_ = 9.8 Hz, 1H, 2- or 3-H); 6.16(dd, *J*_1_ = 4.8 Hz, *J*_2_ = 9.8 Hz, 1H, 2- or 3-H); 6.25 (d, *J* = 10 Hz, 1H, 6-H); 6.86 (d, *J* = 10 Hz, 1H, 7-H); 6.79(s, 1H, 9-OH); 6.89-7.02(m, 3H, Ar-H); 7.22-7.32(m, 1H, Ar-H); 13.19(s, 1H, 10-OH). ^13^C-NMR δ(CDCl_3_): 22.36; 24.94; 25.18; 29.62; 30.37; 35.17; 35.45; 38.34; 71.15; 113.36; 113.64; 113.69; 115.01; 115.30; 123.1; 123.83; 123.87; 123.91; 127.87; 129.88; 130.07; 130.18; 132.76; 134.67; 142.20; 142.29; 143.83; 153.96; 161.30; 161.38; 164.56; 173.75; 19141. IR ν(cm^-1^): 1717.04, 2958.40, 3044.01, 3409.28 HRMS (ESI): calcd. for C_27_H_27_FO_5_ *m/z* [M-1]^+^: 450.18425, found: 450.17984. ***FR58P5b*** (290 mg, 45 % yield), ^1^H-NMR (300 MHz, CDCl_3_) δ:1.38(d, *J* = 7.1 Hz, 3H, 4-CH_3_); 1.60(s, 3H, 8-CH_3_); 1.65(s, 3H, 8-CH_3_); 2.61(t, *J* = 7.3 Hz, 2H, CH_2_); 2.93(t, *J* = 7.7 Hz, 2H, CH_2_); 3.41-3.53(m, 1H, CH); 3.97-4.06(m,1H, CH); 4.20(dd, *J*_1_ = 7.2 Hz, *J*_2_ = 10.2 Hz, 1H, CHH-O); 4.48(dd, *J*_1_ = 4.2 Hz, *J*_2_ = 10.2 Hz, 1H, CHH-O); 4.6(s,1H, 10-OH); 5.93(dd, *J*_1_ = 4.6 Hz, *J*_2_ = 9.9 Hz, 1H, 2- or 3-H); 6.02(dd, *J*_1_ = 4.8 Hz, *J*_2_ = 9.9 Hz, 1H, 2- or 3-H); 6.25 (d, *J* = 10 Hz, 1H, 7-H); 6.83 (d, *J* = 10 Hz, 1H, 6-H); 6.85-6.98(m, 3H, Ar-H); 7.19-7.26(m, 1H, Ar-H); 13.28(s, 1H, 9-OH). ^13^C-NMR δ(CDCl_3_): 22.43; 25.09; 25.28; 29.62; 30.21; 30.63; 34.34; 35.63; 38.21; 67.37; 113.36; 113.01; 113.02; 113.30; 115.08; 115.37; 121.34; 123.99; 124.03; 126.10; 129.89; 130.00; 131.25; 133.36; 136.73; 142.20; 142.67; 142.99; 143.09; 154.72; 161.11; 161.25; 164.51; 172.72; 191.22. IR ν(cm^-1^): 1728.30, 2963.99, 3036.33, 3209.97 HRMS (ESI): calcd. for C_27_H_27_FO_5_ *m/z* [M-1]^+^: 450.18425, found: 450.18024.

***(9,10-dihydroxy-4,8,8-trimethyl-5-oxo-1,4,5,8-tetrahydroanthracen-1-yl)methyl 3,3-diphenyl propanoate (FR58P6a)*** *and* ***(9,10-dihydroxy-4,5,5-trimethyl-8-oxo-1,4,5,8-tetrahydroanthracen-1-yl)methyl 3,3-diphenyl propanoate (FR58P6b).*** 230 mg (1.14 mmol) of Q1 react with 350 mg (1.14 mmol) of (2*E*,4*E*)-2,4-hexadien-1-yl 3,3-diphenylpropanoate in 10 mL of toluene yielding: ***FR58P6a*** (213 mg, 37 % yield),^1^H-NMR δ(CDCl_3_):1.28(d, *J* = 6.9 Hz, 3H, 4-CH_3_); 1.60(s, 3H, 8-CH_3_); 1.66(s, 3H, 8-CH_3_); 3.18(d, *J* = 8.2 Hz, 2H, CH_2_); 3.47(bs, 1H, CH); 3.70(dd, *J*_1_ = 8.9 Hz, *J*_2_ = 11 Hz, 1H, CHH-O); 4.30(dd, *J*_1_ = 4.2 Hz, *J*_2_ = 11 Hz, 1H, CHH-O); 4.61(t, *J* = 8.1 Hz, 1H, CH); 5.50(dd, *J*_1_ = 5 Hz, *J*_2_ = 9.8 Hz, 1H, 2- or 3-H); 6.05(dd, *J*_1_ = 5 Hz, *J*_2_ = 9.8 Hz, 1H, 2- or 3-H); 6.24 (d, *J* = 10 Hz, 1H, 6-H); 6.84 (d, *J* = 10 Hz, 1H, 7-H); 6.86(s, 1H, 9-OH); 7.16-7.36(m, 10H, Ar-H); 13.16(s, 1H, 10-OH). ^13^C-NMR δ(CDCl_3_): 22.36; 24.91; 25.12; 29.51; 35.05; 38.35; 40.86; 47.14; 71.19; 113.66; 123.42; 123.88; 126.86; 126.88; 127.49; 127.58; 127.81; 128.74; 129.26; 132.48; 134.11; 142.73; 142.77; 143.91; 153.78; 161.44; 173.60; 191.42. IR ν(cm^-1^): 1710.93, 2955.51, 3030.55, 3369.13 HRMS (ESI): calcd. for C_33_H_31_O_5_ *m/z* [M-1]^+^: 507.21715, found: 507.21741. ***FR58P6b*** (242 mg, 42 % yield), ^1^H-NMR δ(CDCl_3_):1.37(d, *J* = 7.1 Hz, 3H, 4-CH_3_); 1.60(s, 3H, 5-CH_3_); 1.66(s, 3H, 5-CH_3_); 3.05(d, *J* = 8.1 Hz, 2H, CH_2_); 3.38-3.50(m, 1H, CH); 4.08(dd, *J*_1_ = 7.4 Hz, *J*_2_ = 10.1 Hz, 1H, CHH-O); 4.39(dd, *J*_1_ = 4 Hz, *J*_2_ = 10.1 Hz, 1H, CHH-O); 4.53(t, *J* = 8 Hz, 1H, CH); 4.59(s, 1H, 10-OH); 5.74(dd, *J*_1_ = 4.8 Hz, *J*_2_ = 10 Hz, 1H, 2- or 3-H); 5.94(dd, *J*_1_ = 5 Hz, *J*_2_ = 10 Hz, 1H, 2- or 3-H); 6.26 (d, *J* = 10 Hz, 1H, 7-H); 6.84 (d, *J* = 10 Hz, 1H, 6-H); 7.13-7.32(m, 10H, Ar-H); 13.24(s, 1H, 9-OH). ^13^C-NMR δ(CDCl_3_): 22.43; 25.06; 25.23; 30.12; 34.23; 38.13; 40.85; 47.01; 67.32; 112.93; 121.28; 123.96; 126.13; 126.46; 126.50; 127.61; 127.63; 128.49; 128.51; 130.84; 133.16; 136.53; 142.54; 143.32; 143.39; 154.87; 160.97; 171.81; 191.42. IR ν(cm^-1^): 1728.30, 2961.09, 3033.44, 3531.19 HRMS (ESI): calcd. for C_33_H_32_O_5_ m/z M^+^: 508.22497, found: 508.22098

**SUPPLEMENTARY FIGURES**


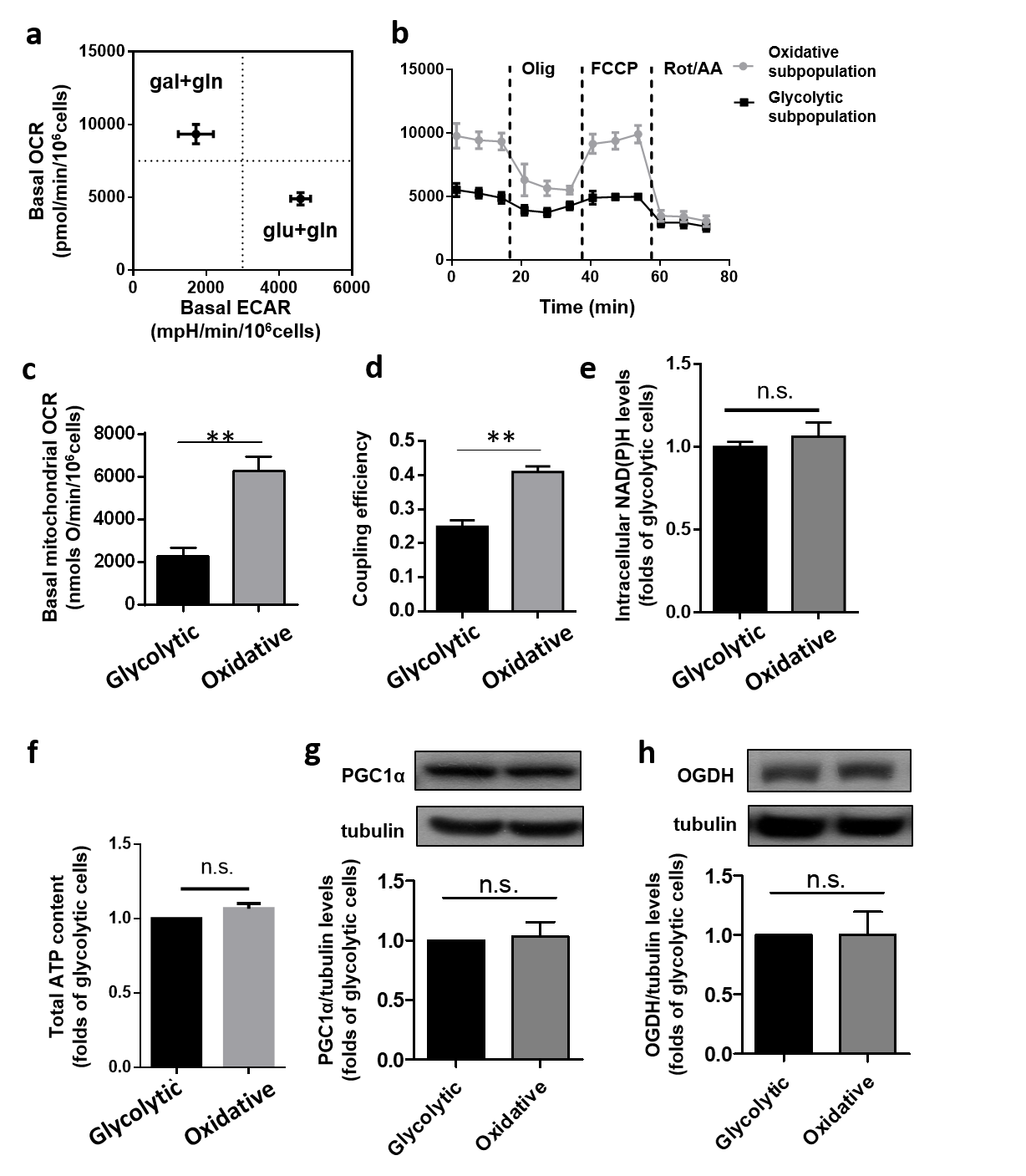


**Supplementary Figure S1:** **(a-b)** Bioenergetic prolife of TNBC MDA-MB-231 oxidative and glycolytic subpopulations and **(c-d)** Basal mitochondrial respiration and coupling efficiency (calculated as ATP-driven respiration/Basal mitochondrial respiration). Levels of **(e)** intracellular NAD(P)H, **(f)** ATP, **(g)** PGC1α and **(h)** OGDH in oxidative and glycolytic subpopulations. Data shown are the mean ± SEM of three independent experiments. **P<0.01 vs glycolytic subpopulation, n.s.: noy significant.


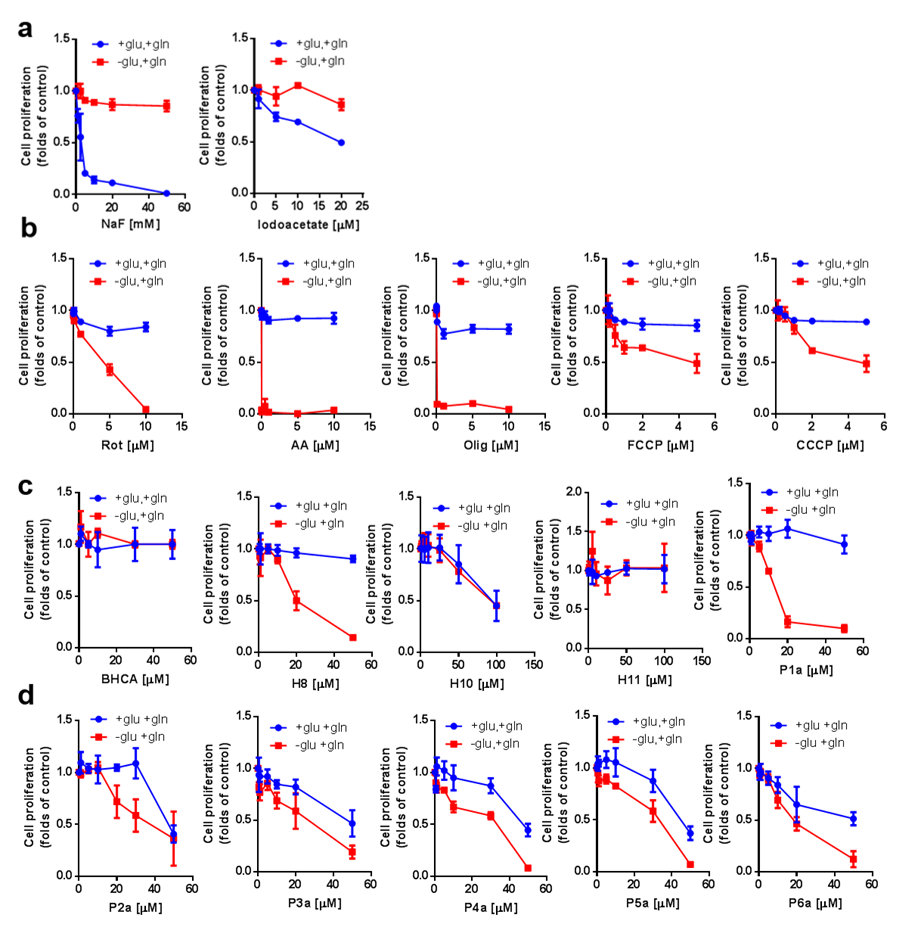


**Supplementary Figure S2:** **(a)** Effect of glycolytic inhibitors, **(b)** mitochondrial ETC inhibitors and uncouplers of OXPHOS and **(c-d)** new compounds on proliferation of MDA-MB-231 subpopulations with glycolytic (blue line) and oxidative phenotypes (red line). Cells were treated with compounds for 48 h and MTT reduction was measured. Data shown are the mean ± SEM of three independent experiments.


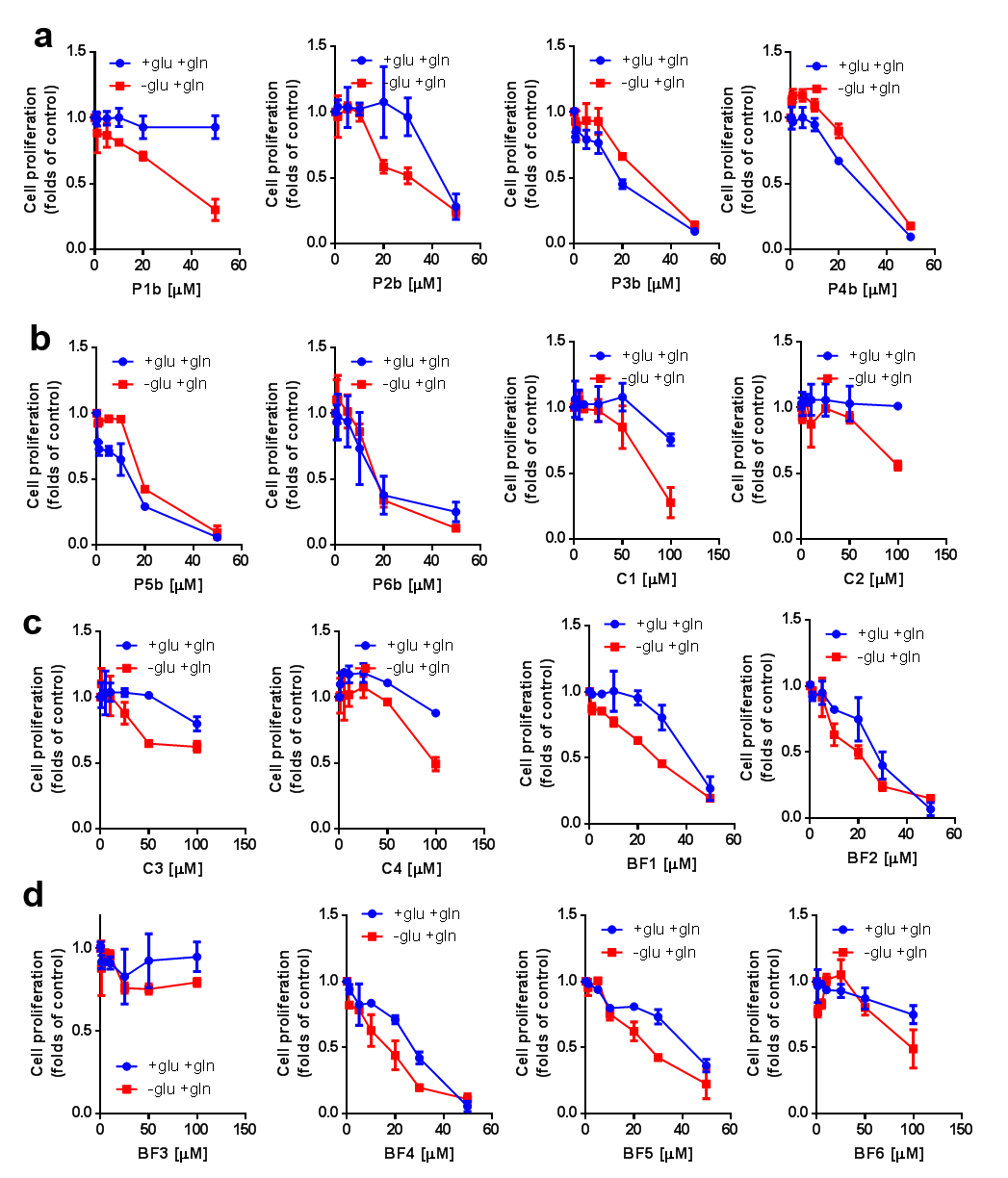


**Supplementary Figure S3:** **(a-d)** Effect of new compounds on proliferation of MDA-MB-231 subpopulations with glycolytic (blue line) and oxidative phenotypes (red line). Cells were treated with compounds for 48 h and MTT reduction was measured. Data shown are the mean ± SEM of three independent experiments.

**
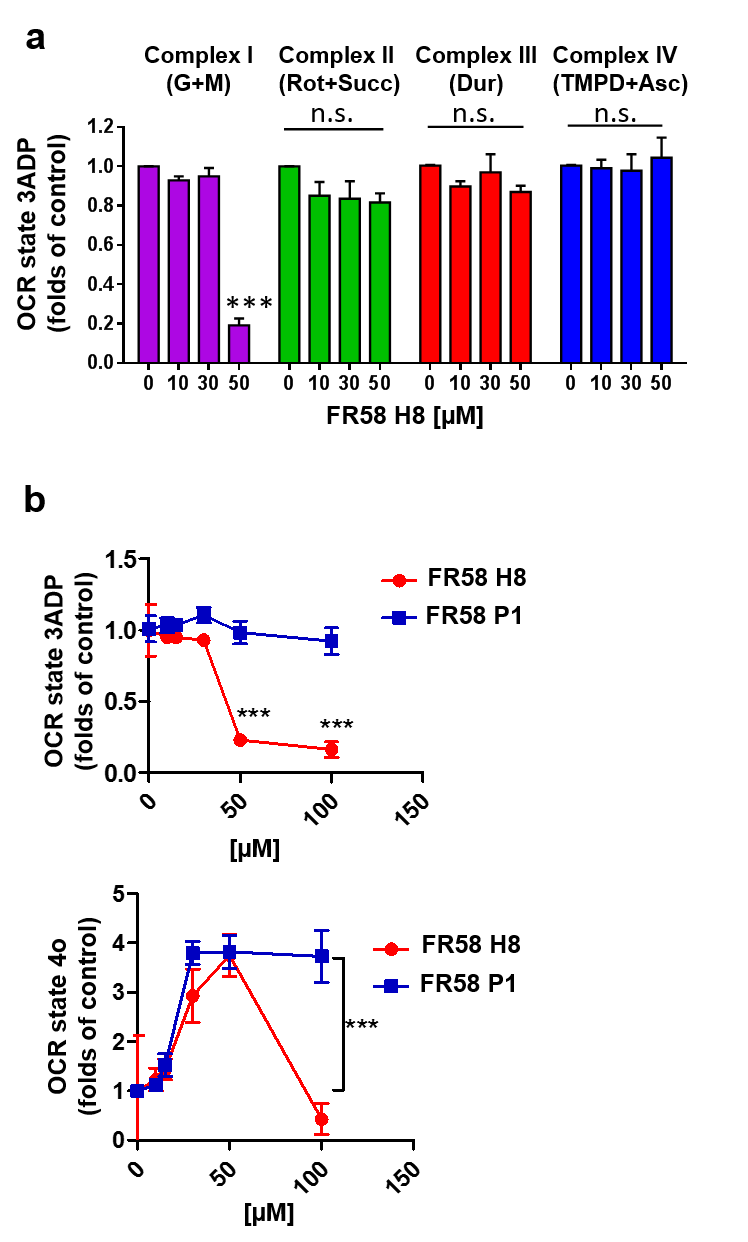
**

**Supplementary Figure S4**. **(a)** Effect of FR58H8 on each respiratory complex dependent-respirations in mitochondria isolated from TA3/Ha murine breast cancer cells. **(b)** Effect of FR58P1a and FR58H8 on respiration of isolated mitochondria in state 3ADP and state 4o. Data shown are the mean ± SEM of three independent experiments. ***p<0.001 vs. Control (DMSO). n.s. not significant. G+M:glutamate plus malate; Rot+Succ: rotenone plus succinate; Dur: duroquinol; TMPD+Asc: N,N,N',N'-Tetramethyl-p-Phenylenediamine plus ascorbate.


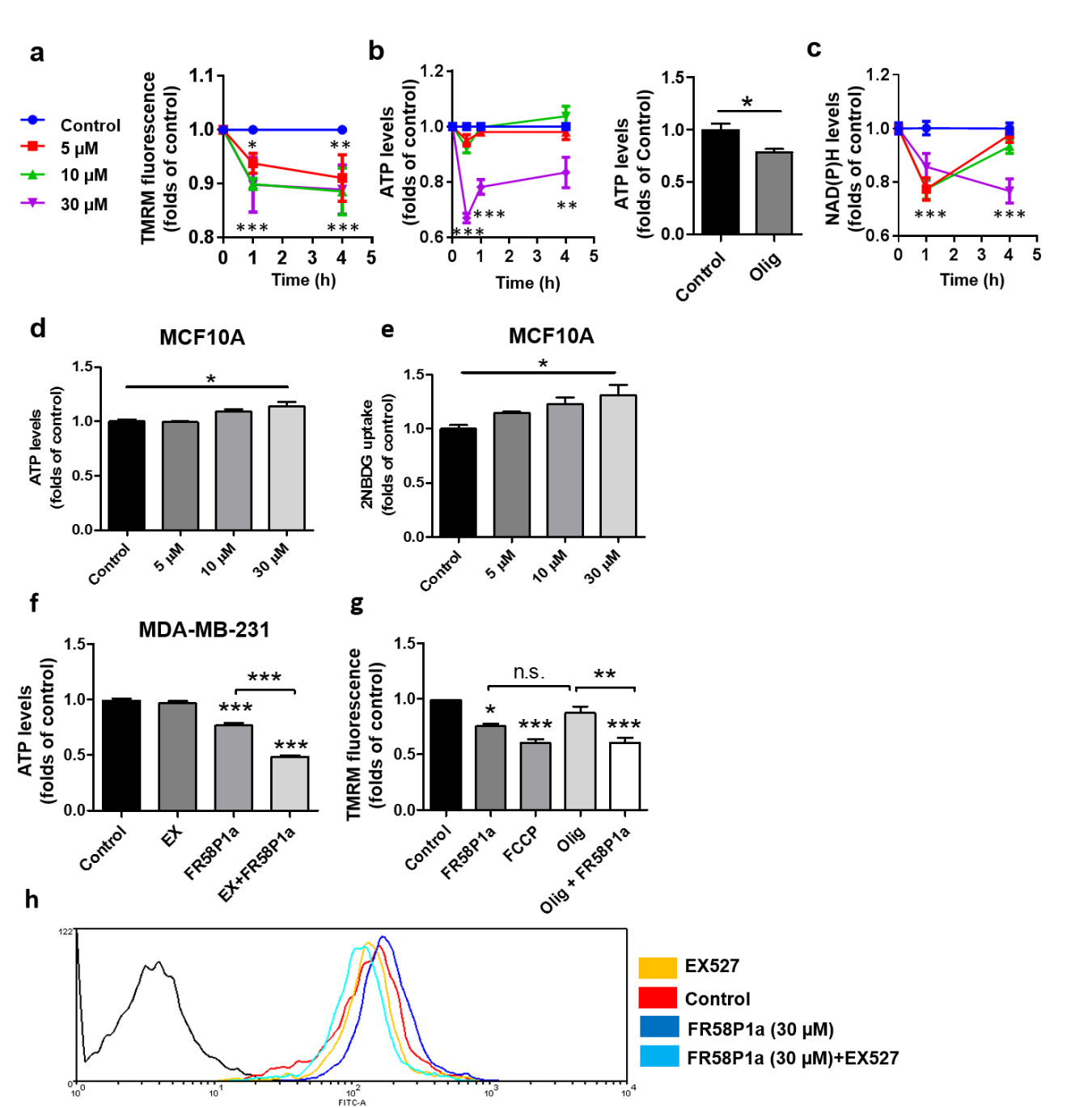


**Supplementary Figure S5**. **(a-c)** Effect of FR58H8 on bioenergetics parameters during 4 h of treatment in MDA-MB-231 cells and **(d-e)** Intracellular ATP levels and 2NBDG uptake of MCF10A cells at 4 h of treatment. Effect of EX527 (10 µM) and oligomycin (2 µM) on **(f)** ATP levels, **(g)** mitochondria membrane potential and **(h)** 2NBDG uptake in MDA-MB-231 cells treated for 4 h with FR58P1a (30 µM). Data shown are the mean ± SEM of three independent experiments. *p<0.05, **P<0.01, ***P<0.001 vs. Control (DMSO).


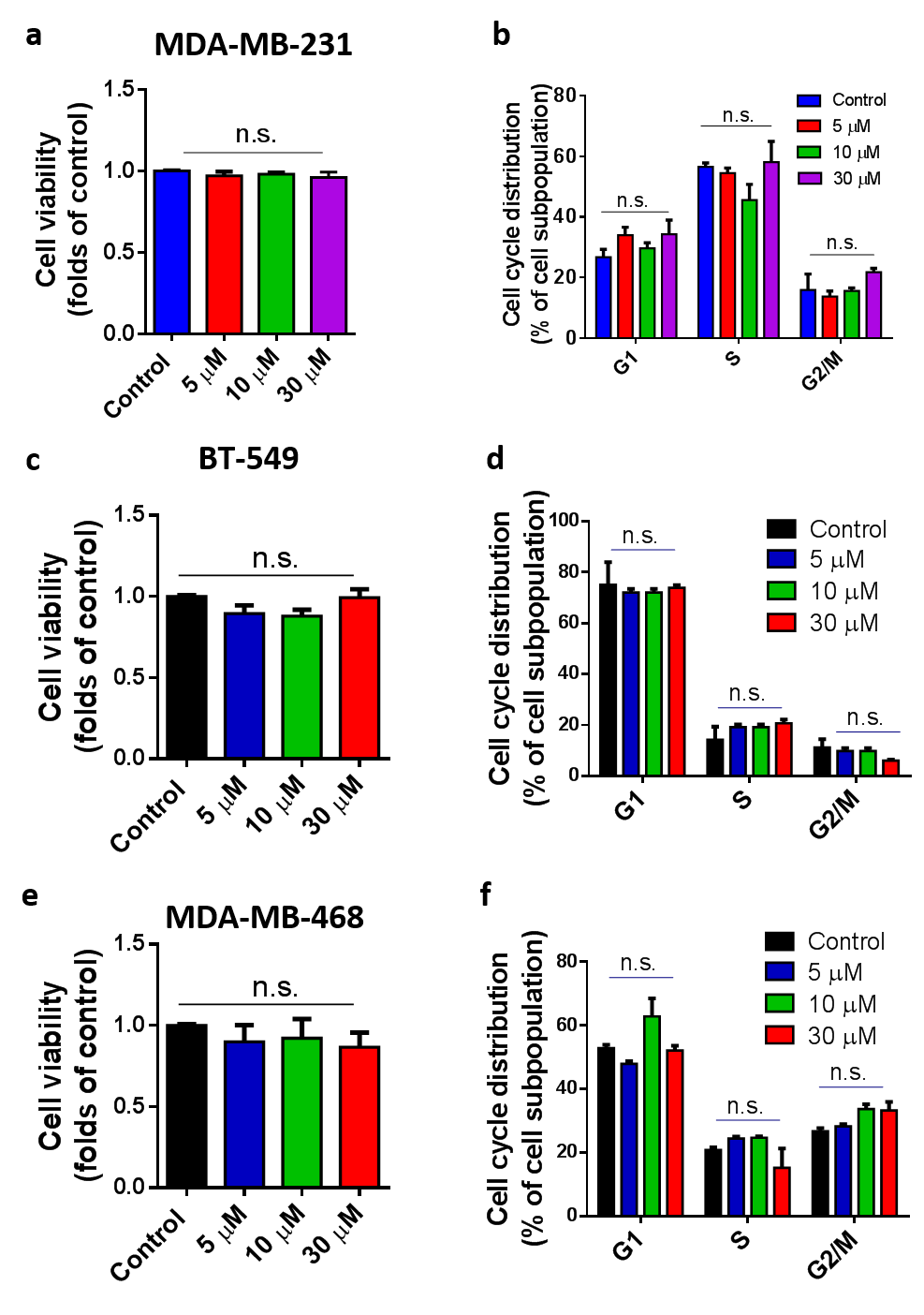


**Supplementary Figure S6**. **Effect of FR58P1a on viability and cell cycle progression of TNBC cells**. **(a-b)** Effect of FR58P1a on viability and cell cycle progression at 48 h of exposure on MDA-MB-231 cells, **(c-d)** BT-549 and **(e-f)** MDA-MB-468 triple negative breast cancer cells. Data shown are the mean ± SEM of three independent experiments. n.s.: not significant vs Control.


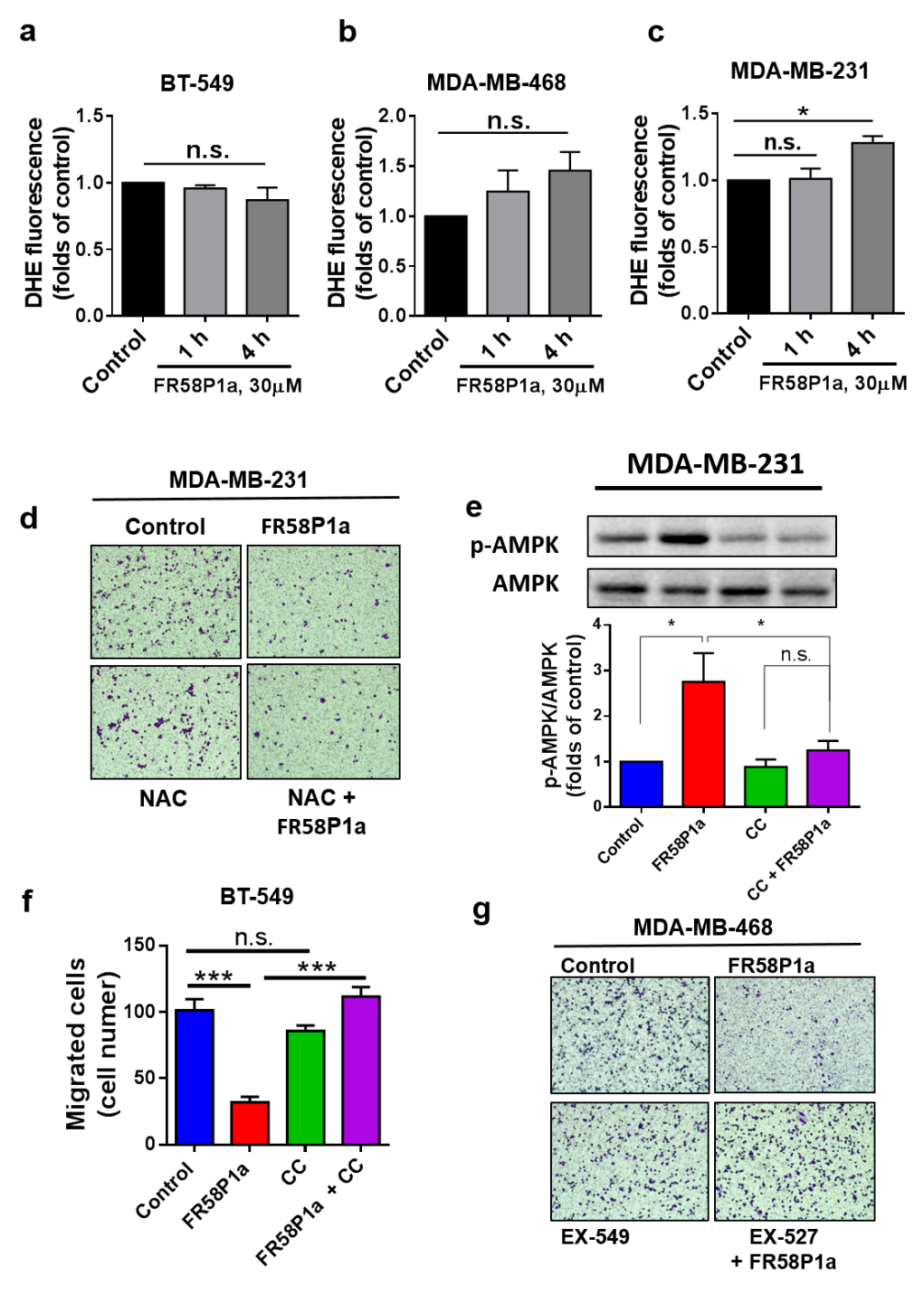


**Supplementary Figure S7**. **Effect of FR58P1a on fibronectin-dependent migration of TNBC cells**. **(a-c)** Effect of FR58P1a on ROS levels of TNBC cells. **(d)** ROS scavenger NAC (4 mM) does not prevent the anti-migratory effect of FR58P1a (30 µM) on MDA-MB-231 cells at 4 h of exposition. (d) 10 µM Compound C prevents the AMPK activation by **FR58P1a;** **(f-g)** CC (10 µM) and EX-527 (10 µM) prevent the anti-migratory effect induced by FR58P1a (30 µM) in TNBC cells. Data shown are the mean ± SEM of three independent experiments. *P<0,05, ***P<0,001 vs Control. n.s.: not significant.


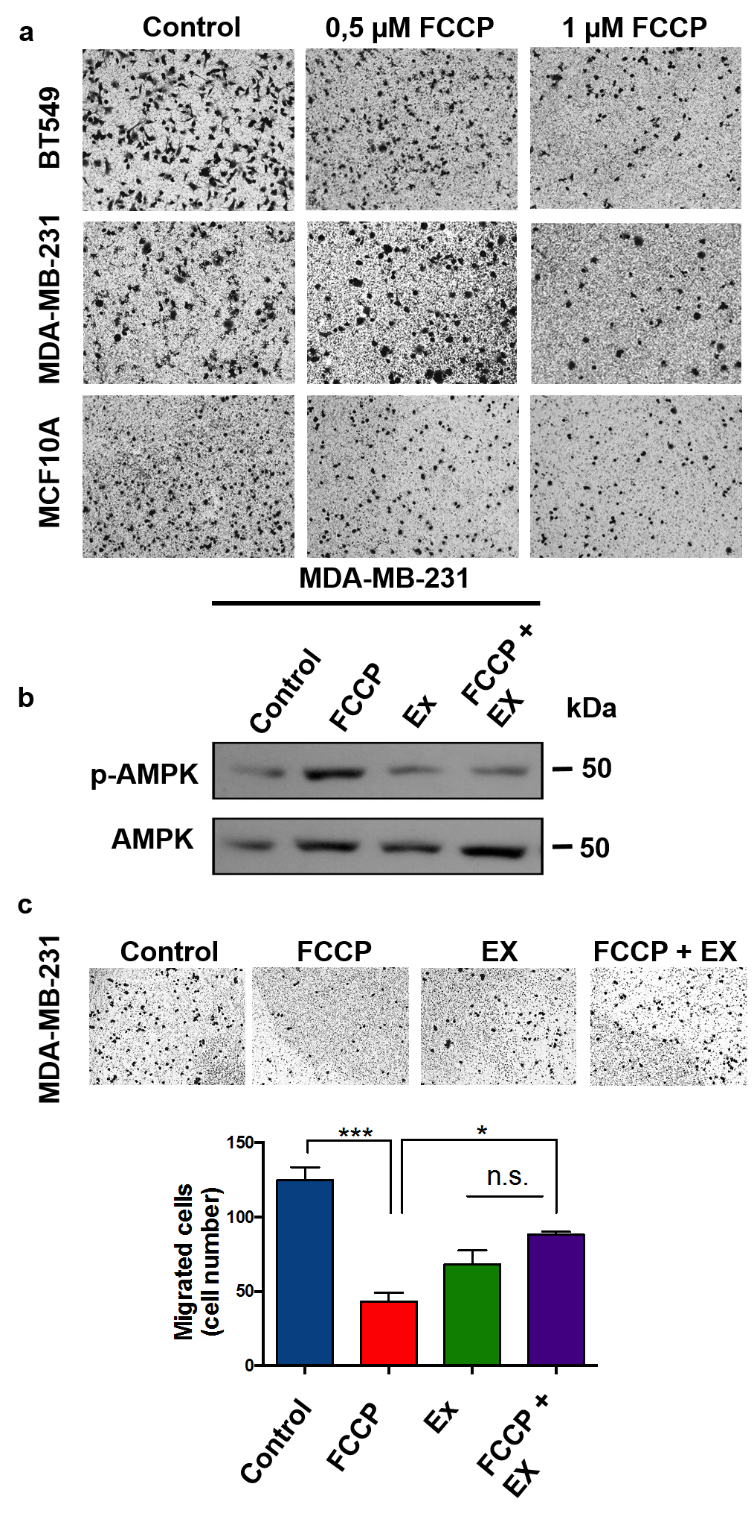


**Supplementary Figure S8. FCCP inhibits fibronectin-dependent migration in a cancer non-selective fashion**. **(a)** representative photographs of the effect of FCCP on the migration in TNBC MDA-MB-231, BT549 and non-tumorigenic MFC10A. **(b)** FCCP activates Sirt1-dependent AMPK and **(c)** this signaling is involved in an anti-migratory effect. Data shown are the mean ± SEM of three independent experiments. *P<0,05, ***P<0,001 vs Control. n.s.: not significant.


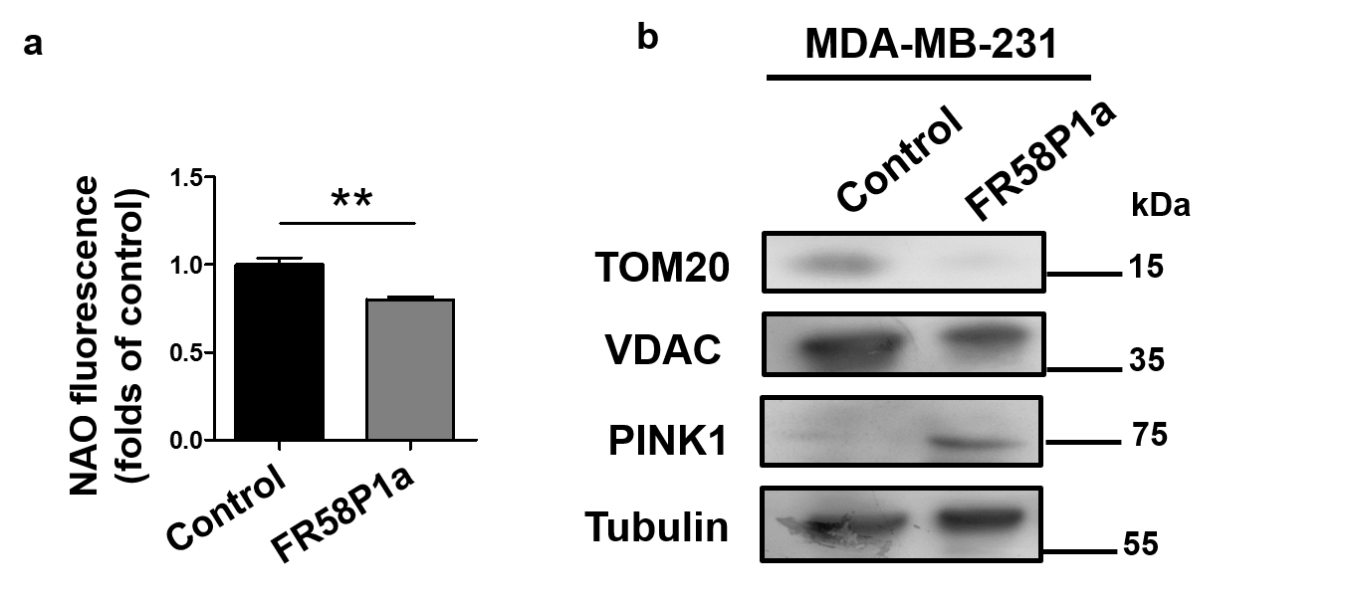


**Supplementary Fig. S9: Prolonged FR58P1a treatment induces changes in mitochondrial composition in MDA-MB-231 cells.** **(a)** Effect of FR58P1a on cardiolipin content and **(b)** expression levels of TOM20, VDAC and PINK1 at 24 h of treatment. Data shown are the mean ± SEM of three independent experiments. **P<0,01 vs Control. n.s.: not significant.


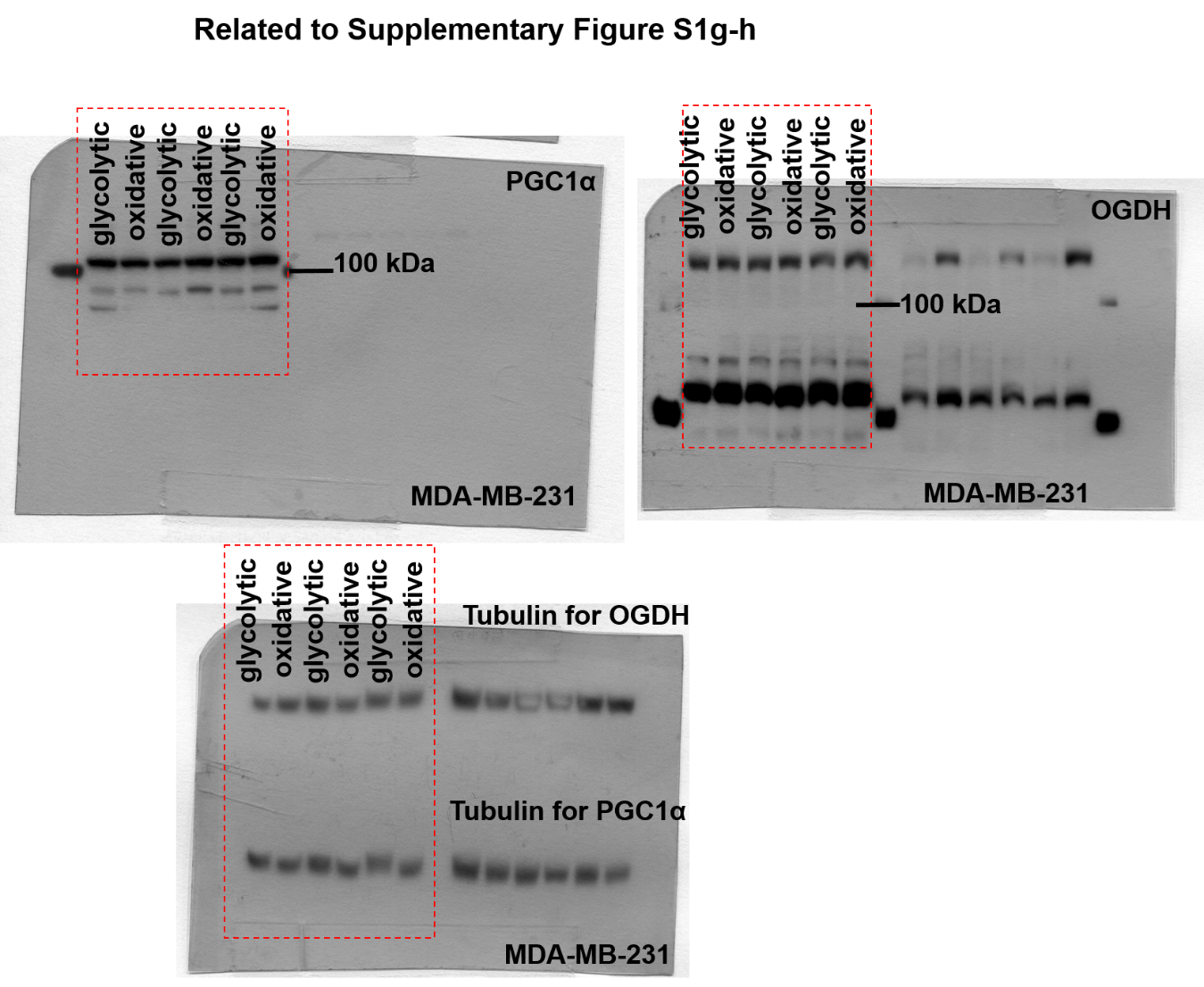


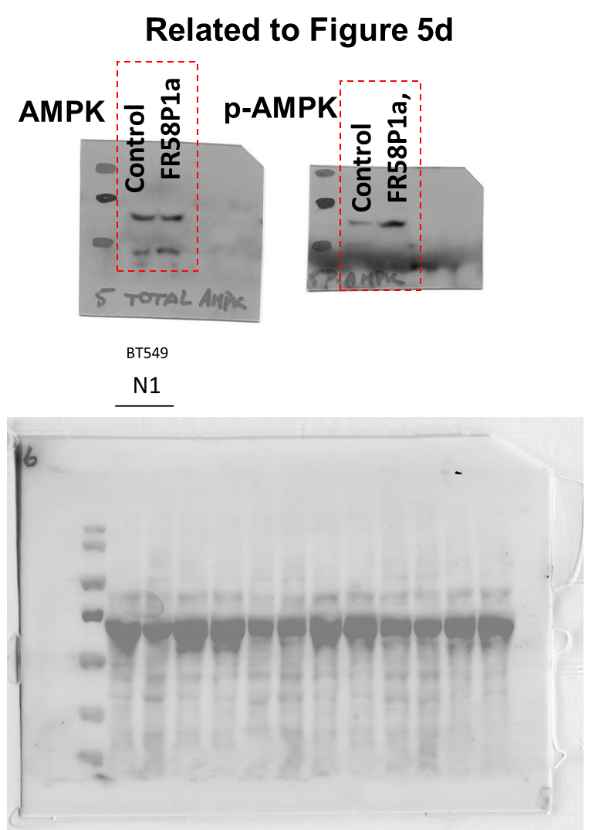


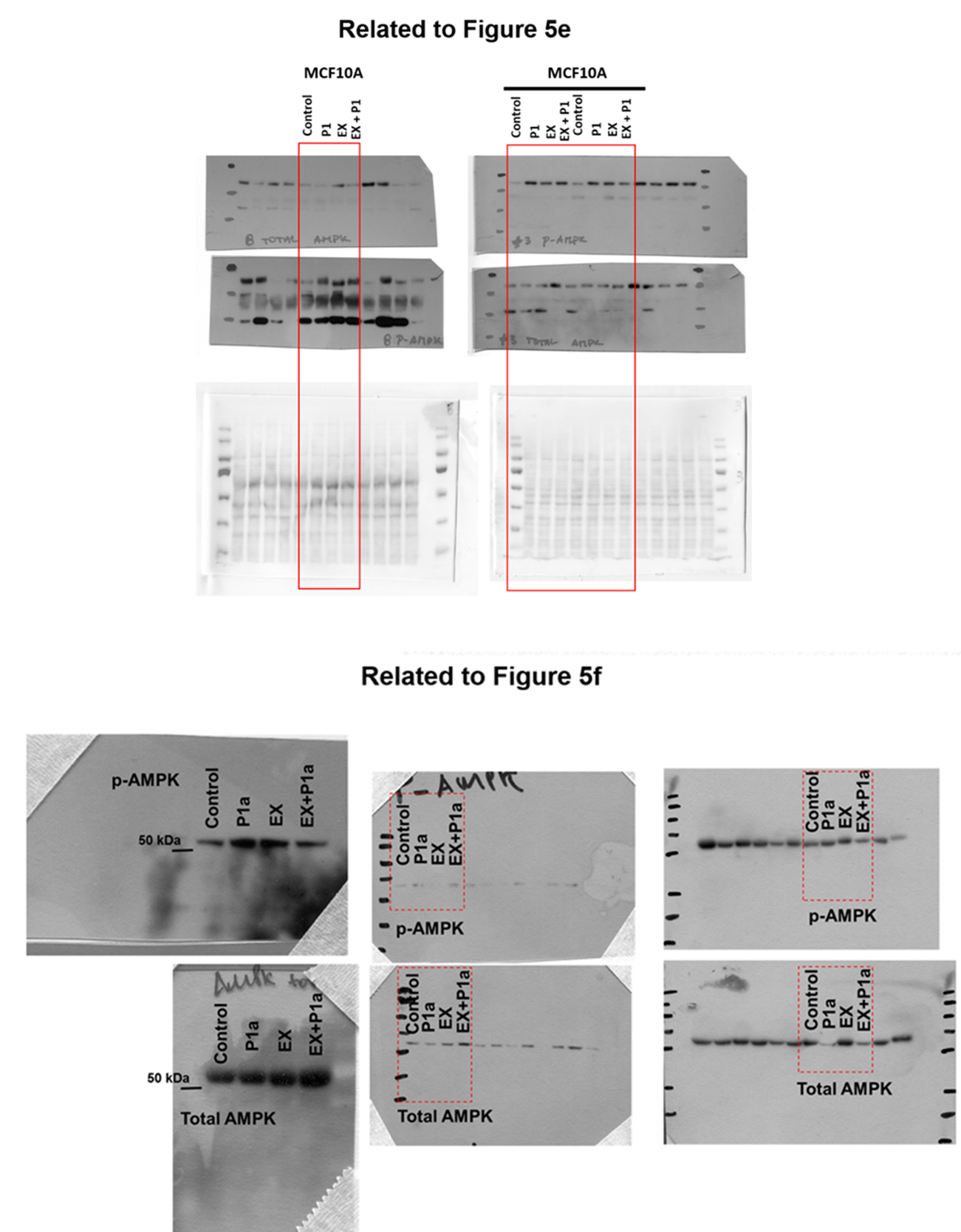


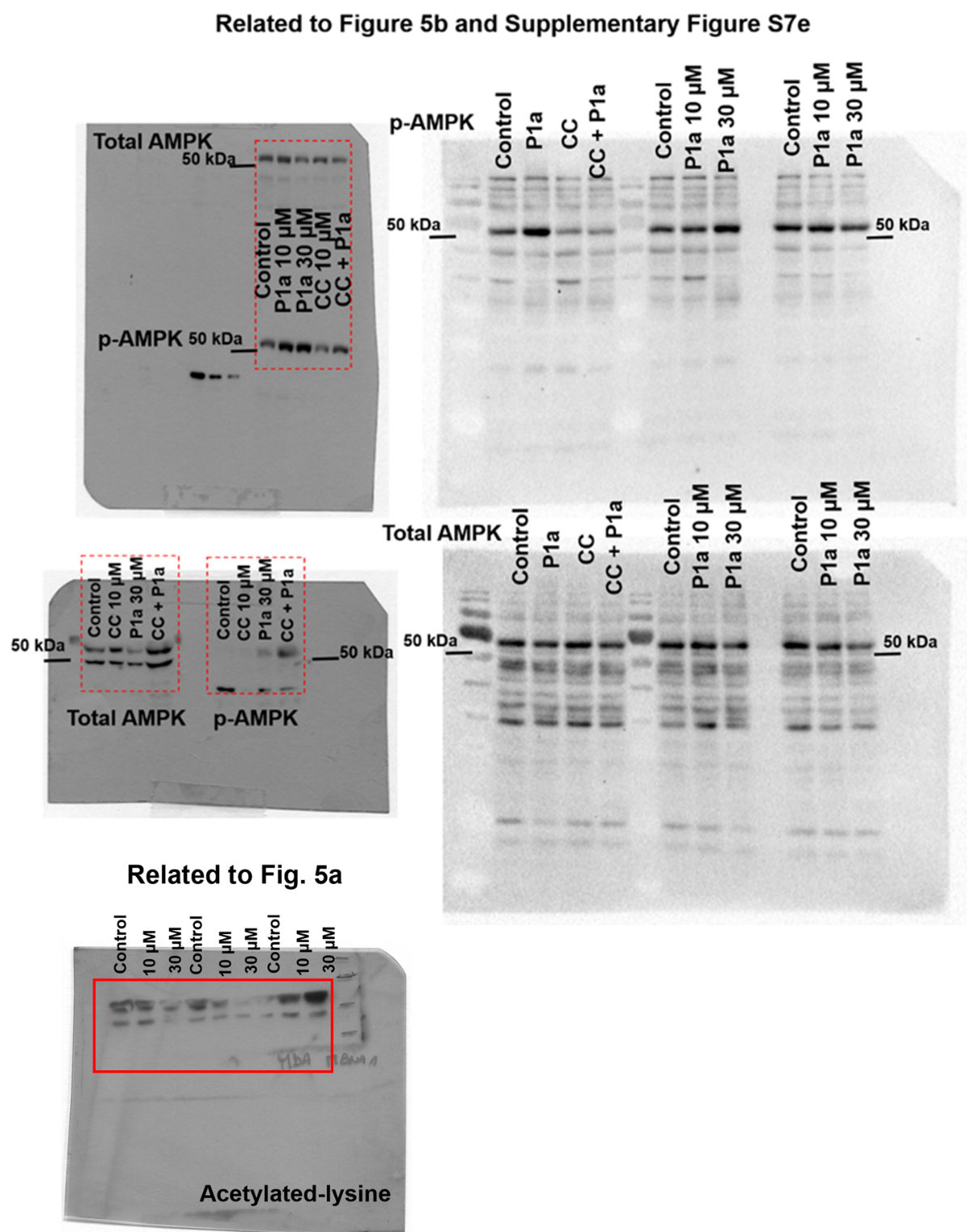


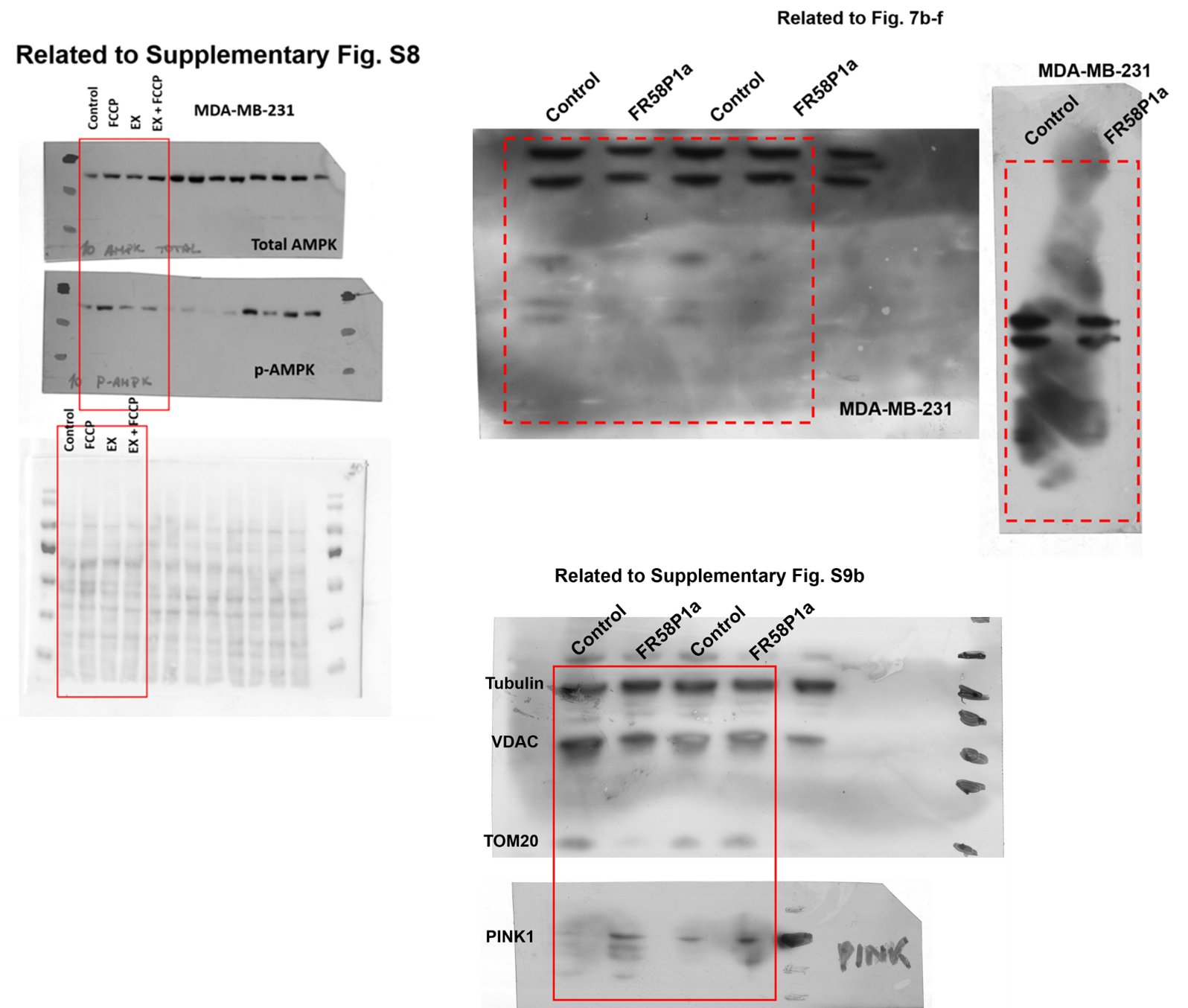


**Supplementary Table S1: Values IC50 and S-values obtained for *ortho*-carbonyl substituted phenolic compounds**

| **Compound** | **IC50 +glu,+gln** | **IC50 -glu,+gln** | **S value [log (IC50 +glu+gln/IC50-glu+gln)]** | |
| --- | --- | --- | --- | --- |
| FR58H8 | 280 µM | 18,97 µM | + | 1,17 |
| FR58H10 | 153.4 µM | 134.7 µM | + | 0.056 |
| FR58H11 | 2.79 mM | 1.15 mM | + | 0.384 |
| **FR58P1a** | **452,1 µM** | **12,32 µM** | **+** | **1,56** |
| FR58P2a | 147 µM | 73.6µM | + | 0.300 |
| FR58P3a | 50,52 µM | 27,06 µM | + | 0,27 |
| FR58P4a | 46,51 µM | 21,95 µM | + | 0,33 |
| FR58P5a | 39,29 µM | 23,35 µM | + | 0,23 |
| FR58P6a | 32.12 mM | 41.28 mM | + | 0,89 |
| FR58P1b | 556.3 µM | 33.58 µM | + | 1.22 |
| FR58P2b | 124.3µM | 48.43µM | + | 0.409 |
| FR58P3b | 67.89 µM | 38.32 µM | + | 0.25 |
| FR58P4b | 56.05 µM | 31.04 µM | + | 0.26 |
| FR58P5b | 52.32 µM | 23.11 µM | + | 0.35 |
| FR58P6b | 50.13 µM | 46.62 µM | + | 0.03 |
| FR58C1 | 660.2 µM | 115.7 µM | + | 0.75 |
| FR58C2 | 846.2 µM | 207.0 µM | + | 0.61 |
| FR58C3 | 697.2 µM | 144.3 µM | + | 0.68 |
| FR58C4 | 401.8 µM | 185.4 µM | + | 0.34 |
| FR58BF1 | 57,82 µM | 24,39 µM | + | 0,37 |
| FR58BF2 | 23,89 µM | 14,4 µM | + | 0,22 |
| FR58BF3 | 89,59 µM | 58,89 µM | + | 0,18 |
| FR58BF4 | 25,87 µM | 13,67 µM | + | 0,28 |
| FR58BF5 | 48,78 µM | 24,36 µM | + | 0,30 |
| FR58BF6 | 173,1 µM | 92,65 µM | + | 0,27 |
| BHA | >500 µM | > 500 µM | n.d. | n.d. |
| Oligomycin | 31,72 µM | 0,0267 µM | + | 3,07 |
| Rotenone | 35,32 µM | 2,61 µM | + | 1,13 |
| Antimicyn-A | 87.5 µM | 0,020 µM | + | 3.64 |
| FCCP | 147,2 µM | 4,189 µM | + | 1,55 |
| CCCP | 273,4 µM | 4,109 µM | + | 1,82 |
| Iodoacetic acid | 19,01 µM | 131,1 µM | - | 0,84 |
| NaF | 2,31 mM | 206,4 mM | - | 1,17 |
|  |  |  |  |  |

**Supplementary Table S2:** List of primers used in this study.

| **Gene/ Description** | **Forward (5'-3')** | **Reverse (5'-3')** | **Ref.** |
| --- | --- | --- | --- |
| Glut1: Solute carrier family 2 (facilitated glucose transporter), member 1 | TGCAGTTCGGCTATAACACTG | GGTGGTTCCATGTTTGATTG | ^19^ |
| Glut4: Solute carrier family 2 (facilitated glucose transporter), member 4 | CTTGGCTCCCTTCAGTTTG | CACGTTGCATTGTAGCTCTG | ^19^ |
| COX-IV isoform1: Mitochondrial cytochrome c oxidase subunit IV | AGAAGGCGCTGAAGGAGAAGGA | CCAGCATGCCGAGGGAGTGA | ^20^ |
| NRF-1: Nuclear respiratory factor-1 | ATGGGCCAATGTCCGCAGTGATGTC | GGTGGCCTCTGATGCTTGCGTCGTCT | ^20^ |
| Cyt C: Cytochrome C | CTTTGGGCGGAAGACAGGTC | TTATTGGCGGCTGTGTAAGAG | ^21^ |
| ATP synt_ATP5Fa1: Mitochondrial ATP synthase alpha chain | TGCAAGGAACTTCCATGCCTC | CGCCCAGTTTCTTCAAGATCAA | ^21^ |
| ANT2: Adenine nucleotide translocator 2 | GCTTGTGTATGATGAAA | AGAAAACTGGTCAGATGAAA | ^22^ |
| ANT3: Adenine nucleotide translocator 3 | TCGAGAAATTCCAGTTGTCTTT | AGAACACGACTTGGCTCCTACA | ^22^ |
| PGC1a: Peroxisome proliferator-activated receptor gamma coactivator 1-alpha | CTCTCCTTGCAGCACCAGAA | CCATCCATGGCTAGTCCTGA | ^23^ |
| GAPDH: Glyceraldehyde-3-phosphate dehydrogenase | CTCTGCTCCTCCTGTTCGAC | ACGACCAAATCCGTTGACTC | ^24^ |

**References**

1 Lou, P. *et al.* Mitochondrial uncouplers with an extraordinary dynamic range. *Biochem J.* **407**, 129-140 (2007).

2 Urra, F. A. *et al.* An ortho-carbonyl substituted hydroquinone derivative is an anticancer agent that acts by inhibiting mitochondrial bioenergetics and by inducing G2/M-phase arrest in mammary adenocarcinoma TA3. *Toxicol Appl Pharmacol.* **267**, 218-227, doi:<http://dx.doi.org/10.1016/j.taap.2012.12.023> (2013).

3 Urra, F. *et al.* Small structural changes on a hydroquinone scaffold determine the complex I inhibition or uncoupling of tumoral oxidative phosphorylation. *Toxicol. Appl. Pharmacol.* **291**, 46-57, doi:10.1016/j.taap.2015.12.005. (2016).

4 Bartolomé, F. & Abramov, A. Measurement of mitochondrial NADH and FAD autofluorescence in live cells. *Methods Mol Biol.* **1264**, 263-270, doi:10.1007/978-1-4939-2257-4_23 (2015).

5 Cárdenas, C. *et al.* Selective vulnerability of cancer cells by inhibition of Ca(2+) transfer from endoplasmic reticulum to mitochondria. *Cell Rep.* **14**, 2313-2324, doi:10.1016/j.celrep.2016.02.030. (2016).

6 Petit, J., Maftah, A., Ratinaud, M. & Julien, R. 10N-nonyl acridine orange interacts with cardiolipin and allows the quantification of this phospholipid in isolated mitochondria. *Eur J Biochem.* **209**, 267-273. (1992).

7 Yamamoto, N. *et al.* Measurement of glucose uptake in cultured cells. *Curr Protoc Pharmacol.* **71**, 1-26., doi:10.1002/0471141755.ph1214s71 (2015).

8 Pfaffl, M. A new mathematical model for relative quantification in real-time RT-PCR. *Nucleic Acids Res.* **29**, e45., doi:10.1093/nar/29.9.e45 (2001).

9 Talke, I., Hanikenne, M. & Krämer, U. Zinc-dependent global transcriptional control, transcriptional deregulation, and higher gene copy number for genes in metal homeostasis of the hyperaccumulator *Arabidopsis halleri*. *Plant Physiol.* **142**, 148-167., doi:10.1104/pp.105.076232 (2006).

10 Ramakers, C., Ruijter, J., Deprez, R. & Moorman, A. Assumption-free analysis of quantitative real-time polymerase chain reaction (PCR) data. *Neurosci Lett.* **339**, 62-66, doi:10.1016/S0304-3940(02)01423-4. (2003).

11 Araya-Maturana, R. *et al.* Effects of 9,10-dihydroxy-4,4-dimethyl-5,8-dihydro-1(4H)-anthracenone derivatives on tumor cell respiration. *Bioorg. Med. Chem.* **14**, 4664-4669, doi:<http://dx.doi.org/10.1016/j.bmc.2006.02.011> (2006).

12 Araya-Maturana, R., Cassels, B. K., Delgado-Castro, T., Valderrama, J. A. & Weiss-López, B. E. Regioselectivity in the Diels-Alder reaction of 8,8-dimethylnaphthalene-1,4,5(8H)-trione with 2,4-hexadien-1-ol. *Tetrahedron* **55**, 637-648, doi:<http://dx.doi.org/10.1016/S0040-4020(98)01083-7> (1999).

13 Araya-Maturana, R. *et al.* Effects of 4,4-Dimethyl-5,8-dihydroxynaphtalene-1-one and 4,4-Dimethyl-5,8-dihydroxytetralone derivatives on tumor cell respiration. *Bioorg Med Chem.* **10**, 3057-3060, doi:<http://dx.doi.org/10.1016/S0968-0896(02)00154-2> (2002).

14 Dobado, J. A. *et al.* NMR assignment in regioisomeric hydroquinones. *Magn Reson Chem.* **49**, 358-365, doi:10.1002/mrc.2745 (2011).

15 Mendoza, L. *et al.* In Vitro Sensitivity of Botrytis cinerea to Anthraquinone and Anthrahydroquinone Derivatives. *J Agric Food Chem.* **53**, 10080-10084, doi:10.1021/jf0511749 (2005).

16 Vega, A., Ramirez-Rodriguez, O., Martinez-Cifuentes, M., Ibanez, A. & Araya-Maturana, R. 8,8-Diethyl-1,4,5,8-tetrahydronaphthalene-1,4,5-trione. *Acta Cryst.* **65**, o345 (2009).

17 Millas-Vargas, J. *et al.* Cyclic O,N acetals derived from acylhydroquinones: Synthesis, anti-proliferative and anti-migratory effects on breast cancer cells. *Molecules.* **In submittion.** (2018).

18 Viglianisi, C., Bartolozzi, M., Pedulli, G., Amorati, R. & Menichetti, S. Optimization of the antioxidant activity of hydroxy-substituted 4-thiaflavanes: a proof-of-concept study. *Chemistry.* **17**, 12396-12404, doi:10.1002/chem.201101146 (2011).

19 Liemburg-Apers, D., Wagenaars, J., Smeitink, J., Willems, P. & Koopman, W. Acute stimulation of glucose influx upon mitoenergetic dysfunction requires LKB1, AMPK, Sirt2 and mTOR-RAPTOR. *J Cell Sci.* **129**, 4411-4423, doi:10.1242/jcs.194480 (2016).

20 Rossmeisl, M. *et al.* Expression of the uncoupling protein 1 from the aP2 gene promoter stimulates mitochondrial biogenesis in unilocular adipocytes in vivo. *Eur J Biochem.* **269**, 19-28 (2002).

21 LeBleu, V. *et al.* PGC-1α mediates mitochondrial biogenesis and oxidative phosphorylation in cancer cells to promote metastasis. *Nat Cell Biol.* **10**, 1-15, doi:10.1038/ncb3039 (2014).

22 Desquiret, V. *et al.* Dinitrophenol-induced mitochondrial uncoupling in vivo triggers respiratory adaptation in HepG2 cells. *Biochim Biophys Acta.* **1757**, 21-30, doi:10.1016/j.bbabio.2005.11.005 (2006).

23 Battiprolu, P. *et al.* Metabolic stress-induced activation of FoxO1 triggers diabetic cardiomyopathy in mice. *J Clin Invest.* **122**, 1109-1118., doi:10.1172/JCI60329 (2012).

24 Jiang, Z., Wang, M., Xu, J. & Ning, Y. Hypoxia promotes mitochondrial glutamine metabolism through HIF1α-GDH pathway in human lung cancer cells. *Biochem Biophys Res Commun.* **438**, 32-38., doi:10.1016/j.bbrc.2017.01.015. (2017).
